# Growth patterns from birth to 24 months in Chinese children: a birth cohorts study across China

**DOI:** 10.1186/s12887-018-1328-z

**Published:** 2018-11-05

**Authors:** Fengxiu Ouyang, Fan Jiang, Fangbiao Tao, Shunqing Xu, Yankai Xia, Xiu Qiu, Jun Zhang

**Affiliations:** 1 Ministry of Education and Shanghai Key Laboratory of Children’s Environmental Health, Xinhua Hospital, Shanghai Jiao Tong University School of Medicine, 1665 Kong Jiang Road, Shanghai, 200092 China; 2 Department of Developmental and Behavioral Pediatrics, Shanghai Pediatric Transitional Institution, Shanghai Children’s Medical Center affiliated with Shanghai Jiao Tong University School of Medicine, Shanghai, 200127 China; 3 School of Public Health, Anhui Medical University, Hefei, 230032 China; 4 Key Laboratory of Environment and Health (HUST), Ministry of Education & Ministry of Environmental Protection, and State Key Laboratory of Environmental Health (Incubation), School of Public Health, Tongji Medical College, Huazhong University of Science and Technology, Wuhan, 430074 Hubei China; 5 School of Public Health, Nanjing Medical University, Nanjing, 211166 China; 6 Division of Birth Cohort Study, Guangzhou Women and Children’s Medical Center, Guangzhou Medical University, Guangzhou, 510000 China

**Keywords:** Growth standards, Chinese children, Infancy

## Abstract

**Background:**

Assessment of child growth is important in detecting under- and over-growth. We aimed to examine the growth patterns of healthy Chinese infants from birth to 24 months.

**Methods:**

This study was based on six recent birth cohorts across China, which provided data (from 2015) on 4251 children (2174 boys, 2077 girls) who were born at term to mothers without gestational or preexisting diabetes, chronic hypertension, preeclampsia, or eclampsia. Analyses were performed using 28,298 longitudinal anthropometric measures in 4251 children and the LMS method to generate smoothed Z-score growth curves, which were compared to the WHO growth standards (which are based on data from 2003) and current Chinese growth references (which are based on data from 2005).

**Results:**

Most (80.3%) of mother had college education or more, and maternal smoking was rare (0.4%). Compared to the WHO longitudinal growth standards for children aged 0 to 2 years, the growth references from this longitudinal study (length-, weight-, head circumference-, BMI-for-age, and weight-for-length) were significantly higher, for boys and girls; Specifically, the median length-, weight-, head circumference-, BMI-for-age, and weight-for-length was on average 0.9 (range 0.2–1.3) cm, 0.51 (range 0.09–0.74) kg, 0.17 (range − 0.24 to 0.37) cm, 0.70 (range 0.01 to 0.92) kg/m^2^, and 0.43 (range 0.01 to 1.07) kg higher in Chinese boys, and 1.3 (range 0.5–1.9) cm, 0.73 (range 0.10–0.91) kg, 0.45 (range 0.15–0.62) cm, 0.7 (range 0.0 to 1.0) kg/m^2^, and 0.42 (range 0.00 to 0.64) kg greater in Chinese girls, respectively. Compared to the current China cross-sectional growth references (based on data from a decade ago), growth references from this study were also higher, but the difference was less than that between growth references of this study and WHO growth standards.

**Conclusions:**

This recent multicenter prospective birth cohort study examined early growth patterns in China. The new growth curves represent the growth patterns of healthy Chinese infants evaluated longitudinally from 0 to 24 months of age, and provide references for monitoring growth in early life in modern China that are more recent than WHO longitudinal growth standards from other countries and previous cross-sectional growth references for China.

**Electronic supplementary material:**

The online version of this article (10.1186/s12887-018-1328-z) contains supplementary material, which is available to authorized users.

## Background

The assessment of child growth is important in detecting under- and over-growth, which can provide information for timely intervention. The first 1000 days of life (from conception to 2 years of age) is a period of rapid growth and development, and vulnerable to nutritional and environmental influences [1]. Identifying normal child growth patterns is of fundamental importance in growth assessment.

Both the World Health Organization (WHO) growth standards [2] and the China growth references [3] are being applied in China. The WHO growth standards for children aged 0 to 24 months were constructed based on longitudinal data of children (*n* = 882) by using selection criteria of having socioeconomic conditions favorable to growth and having access to breastfeeding support (for qualifying as “standard”) from the WHO Multicenter Growth Reference Study (MGRS) conducted in six countries from 1997 to 2003 (without a site in China). The China growth charts were constructed from a large (*n* = 44,250) cross-sectional study based on stratified random sampling of children in nine cities of China, which was conducted from May to October in 2005 [3]. Comparison of the growth curves over the restricted range of ages from 0 to 2 years indicated the reference for China was significant higher for BMI for boys and girls. However, the comparisons were complicated by differences in inclusion/exclusion criteria (for the WHO sample, strict criteria about known constraints on growth and cooperation with feeding recommendations, which led to over 80% of mother-infant pairs being ineligible; for the China sample, multistage stratified cluster sampling was used based on urban/suburban areas, districts, and community, with several exclusion criteria), as well as by differences in the design of the studies (longitudinal for the WHO study and cross-sectional for the study in China). The difference between China growth references and WHO growth standards could have been an artifact, so confirmation study is warranted.

Historically, in some circumstances, secular trends of height have occurred from one generation to the next generation [4]. China has a diverse population, environment, dietary habits and tradition, and it is going through rapid modernization and urbanization. Early child growth has drawn much attention since these factors may affect growth. China started the 1st National Survey on the Physical Growth and Development of Children (NSPGDC) in the nine cities of China in 1975, and conducted the survey every 10 years from 1975 to 2005 to address possible secular trends, with the most recent data (from 2005) providing the current references for growth in China [3] (but in need of a 10-year update in 2015). Longitudinal data from a sample with stricter inclusion/exclusion criteria would provide a better comparison to the WHO standards. A small cohort [5] recruited in 2007 (*n* = 1531 retained up to 1 year of age) with strict WHO criteria applied showed significant differences (heavier in weight, longer in length, and bigger in head circumference) compared to WHO standards, as well as compared to the current cross-sectional references, which showed similar differences (except for the 97th percentiles that were lower rather than higher).

Long-term follow-up data has enormous value in evaluating the optimal individual growth trajectory, which may not be captured by cross-sectional data [3, 6]. Between 2012 and 2014, six longitudinal birth cohort studies were launched in China. A number of common exposures shared by all cohorts were collected and common outcomes were observed, which formed the foundation of China Birth Cohort Consortium (CBCC). This collaboration provided, for the first time in China, longitudinal growth data from birth cohorts from various regions of the country, but it still is a convenience sample from an efficient combination of cohorts.

This report examines growth patterns from birth to 24 months in Chinese children by pooling the individual level anthropometric follow-up measures from CBCC. The growth references from the 2015 CBCC will be used for comparison to the 2006 WHO longitudinal growth standards and the 2005 China cross-sectional growth references to provide an update on how healthy infants are growing in modern China.

## Methods

### Study population and data collection

This study used data from six birth cohorts of CBCC which were located at Shanghai (2 cohorts), Anhui, Guangdong, Hubei, and Jiangsu Provinces and were initiated between 2012 and 2014 (Additional file 1: Table S1_1 and S1_2). Additional file 1: Table S1_2 presents the study objective of each of the 6 cohorts. The original aims of these prospective cohorts were to study the environmental, genetic and behavioral factors during pregnancy and in early childhood, and their effects on pregnancy outcomes, fetal and child growth and development, and risks of diseases. Pregnant women were recruited at hospitals when they came for their routine prenatal care visits.

Weight, length, head circumference, and gestational age at birth were obtained from obstetrical medical records. Child anthropometric measurements including weight, length, and head circumference were conducted by trained study staff or trained pediatric nurses in maternal and child health care centers according to the WHO protocol at 7 targeted ages (42 days, 3, 6, 9, 12, 18 and 24 months; http://www.who.int/childgrowth/training/en/). Recumbent length on infants was measured with infant head position in the Frankfort Vertical Plane, and the soles of the feet flat on the moveable footboard. The cohort staffs were trained by group-watching WHO training video course on weight, length, and head circumference. The pediatric nurse measurements were made as routine care was provided. Infant age was calculated by date at measurement minus date of birth. Feeding type in the first 6 months was classified into three types: exclusive breastfeeding, mixed feeding (i.e., combination of breastfeeding and formula feeding), and exclusive/only formula feeding [7]. Infant passive smoking exposure was defined by the mother or father smoking, or for anyone else living in the home smoking. The diagnosis of gestational diabetes mellitus (GDM) in pregnant women was based on the recommendations of International Association of Diabetes and Pregnancy Study Groups (IADPSG) [8].

For this project, we requested each of the six birth cohort studies to contribute longitudinal child growth data of 1000 singleton children from birth to 2 years of age, or maximum number available at the time of our data request in July, 2016. Two cohorts contributed child follow-up measurements up to 12 months due to later starting date (2014) or child follow-up schedule (Additional file 1: Table S1). The inclusion criteria included singleton live births. The exclusion criteria included: (1) infants born with congenital malformations; (2) pregnancy conceived by assisted reproductive technologies (ART); (3) women with medical complication of sexually transmitted diseases (syphilis, HIV infection, and AIDS); (4) women with pre-existed diabetes. There were 5152 mother-child pairs, which provided a sample almost 6 times greater than the WHO longitudinal cohort from 2003 and over 3 times greater than the previous China longitudinal cohort from 2007. While birth cohort studies used better trained personnel for the growth assessments, more observations can also offset “imprecise observations”.

Among the 5152 mothers, 672 had GDM, 213 had preterm deliveries (gestational age < 37 weeks), and 71 had hypertensive disorders in pregnancy. Among the remaining 4258, 7 had missing data on infant sex. To generate the growth references, we used data from 4251 normal term-born children and excluded children of mothers with GDM, hypertensive disorders in pregnancy (e.g., chronic hypertension, gestational hypertension, preeclampsia and eclampsia),children born preterm to avoid the potential influences of known prenatal risk factors [10–12],and children with missing data on sex.

### Statistical analysis

We used the LMS method to fit smooth z-score curves for length, weight, head circumference and BMI according to age, and for weight according to length respectively in normal term-born healthy children, stratified by infant sex. [13] The three curves of median (M), coefficient of variation (S) and skewness (L, which is expressed as a Box-Cox power) across age/or length were fitted as cubic splines by using maximum penalized likelihood [13]. The z-score of child growth measures y (length, weight, head circumference and BMI) at time t (or length t, for weight-for-length) was calculated from the smooth curve L(t), M(t), and S(t) by the formula:

$$ z=\frac{{\left[y/M(t)\right]}^{L(t)}-1}{L(t)S(t)},\mathrm{if}\kern0.5em L\left(\mathrm{t}\right)\ne 0;\kern0.5em z=\frac{\log \left[y/M\left(\mathrm{t}\right)\right]}{S\left(\mathrm{t}\right)},\mathrm{if}\kern0.5em L\left(\mathrm{t}\right)=0 $$

By using the maximum penalized likelihood and LMS method, all available data of infants from birth to 27 months, including those followed up to 12 months were able to be used to estimate the smoothing parameters and generate the smoothed curves [9, 13]. The age-based references were truncated at 24 completed months to avoid the right-edge effect [14]. We compared z-scores of 0, ±2, and ± 3 for the growth measures in this study with the WHO standards (http://www.who.int/childgrowth/standards/en/), and the China 2005 references for children aged 0 to 2 years [3], both of which were constructed using similar LMS methods for smoothing procedures [3, 14]. The two-sided t-test was used to test statistical significance of the difference at a *p* < 0.05. The growth curves were constructed by using LMSchartmaker Pro version 2.54 software (Medical Research Council, UK).

We also calculated the 3rd, 10th, 50th, 90th and 97th percentiles of all growth measures in both boys and girls by age with subgroup sample size > 100 observations to summarize our data (without using smoothing technique), and compared these percentiles with WHO standards to show the differences. The analyses were conducted by using SAS 9.4 software (SAS Institute, Cary, North Carolina).

## Results

**Fig. 1:**
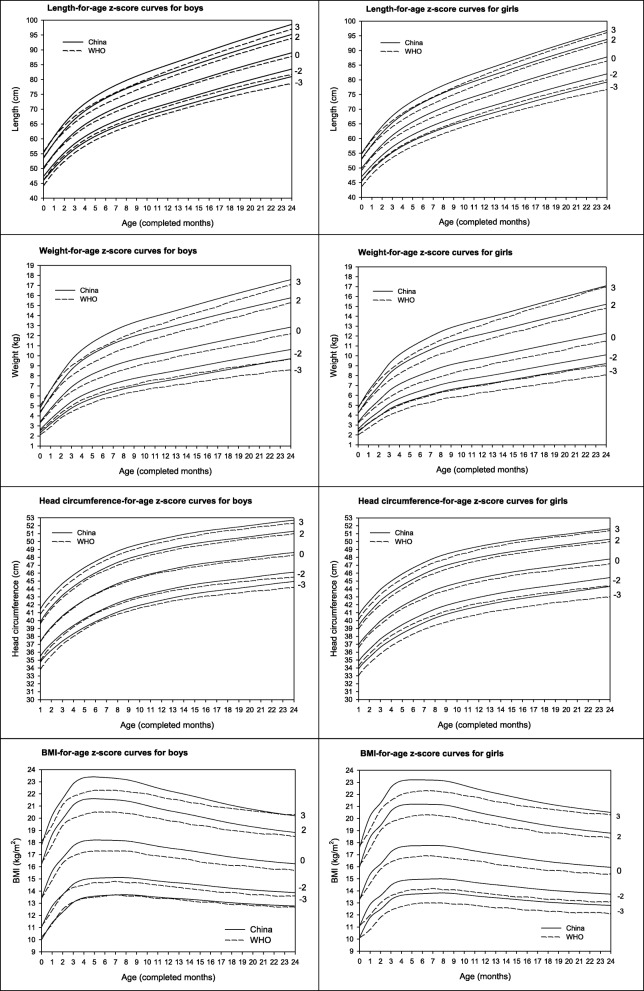
Comparison of growth-for-age z-score curves with WHO standards in boys and girls

This report presented the z-score curves of 4251 children who were born at term to mothers without gestational or preexisting diabetes, chronic hypertension, preeclampsia, or eclampsia. A total of 28,298 anthropometric measures were obtained from ages 0 to 27 months (Additional file 1: Tables S2 and S3). All were urban children. 51.1% were boys and 54.0% were delivered via C-section. The mean maternal and paternal height was 161.4 (SD 4.9) cm and 174.4 (SD 5.3) cm, respectively. Mean (pre-pregnancy) BMI was 20.6 (SD 2.8) kg/m^2^ for mothers and 23.9 (SD 3.3) kg/m^2^ for fathers. As expected, boy infants had greater birthweight, length and head circumference than girl infants (Table 1). Most (80.3%) of mother had college education or more and 98.3% of mother were Han ethnicity. During the first 6 months, most (77.6%) of infants were mixed fed, and 13.4% had exclusive breast-feeding. In the first 2 years, 27.9% of children were exposed to passive-smoking. There was no sex difference for these factors (Table 1). Over the follow-up assessments (see Fig. 1), the children aged 0 to 2 years in this cohort were taller, heavier, and had greater head circumference than the children in the WHO cohort.

**Table 1:** Characteristics of 4251 mothers, fathers and children by child sex

	Infant sex		*p* value
	Boy	Girl	
Sample size	2174	2077	
Maternal factors			
Maternal age (years)	28.7 ± 3.4	28.6 ± 3.5	0.51
Pre-pregnancy weight (kg)	53.8 ± 7.8	53.7 ± 8.1	0.92
Maternal height (cm)	161.3 ± 4.9	161.4 ± 5.0	0.33
Prepregnancy BMI (kg/m^2^)	20.7 ± 2.8	20.6 ± 2.8	0.46
Mother Education			
Junior high school or lower	136(6.3)	135(6.6)	0.90
High school	287(13.4)	266(13.0)	
College or above	1725(80.3)	1641(80.4)	
Mother smoke during pregnancy			
Yes	10(0.5)	7(0.3)	0.53
No	2148(99.5)	2047(99.7)	
Parity			
Primiparous	1958(90.2)	1885(90.9)	0.44
parous	212(9.8)	188(9.1)	
Mode of Delivery			
Vaginal delivery	994(45.8)	957(46.2)	0.79
C-section	1177(54.2)	1115(53.8)	
Paternal factors			
Father age (years)	30.6 ± 4.4	30.6 ± 4.6	0.69
Father height (cm)	174.2 ± 5.2	174.6 ± 5.3	0.04
Father weight (kg)	72.5 ± 11.2	73.1 ± 11.7	0.14
Father BMI (kg/m^2^)	23.9 ± 3.2	23.9 ± 3.3	0.56
Father smoke during mother pregnancy			
Yes	568(32.1)	567(34.0)	0.25
No	1199(67.9)	1101(66.0)	
Infant factors			
Birth weight (g)	3399 ± 404	3309 ± 392	< 0.001
Birth length (cm)	50.2 ± 1.4	49.8 ± 1.3	< 0.001
Birth head circumference (cm)	34.1 ± 1.1	34.0 ± 1.0	0.01
Gestational age (weeks)	39.1 ± 1.0	39.3 ± 1.0	< 0.001
Breastfeeding Type (0–6 months)			
Formula feeding	168(8.7)	172(9.4)	0.36
Exclusive Breastfeeding	252(13.0)	252(13.7)	
Mixed feeding	1518(78.3)	1412(76.9)	
Children passive smoking			
No	1187(72.7)	1125(71.5)	0.44
Yes	445(27.3)	448(28.5)	

Data were presented as mean ± SD, and *n* (%)

χ^2^ test for categorical variables and t-test for continuous variables

### Length-for-age

Table 2 presents the growth references of length-for-age at 0, ±1, ±2, and ± 3 SD in our study. In comparison with the corresponding WHO growth standard from 0 to 24 months of age, the median length-for-age was on average 0.9 cm (range 0.2–1.3 cm) higher in Chinese boys, and 1.3 cm (range 0.5–1.9 cm) higher in Chinese girls (Fig. 1). Similarly, for z-score of − 2 (i.e. the cutoffs for defining stunting), child length was on average 1.1 cm taller (range 0.8–1.8 cm) in Chinese boys and 1.6 (range 1.1–2.0) cm taller in Chinese girls than the corresponding sex-specific WHO curves. Likewise, for z-score of − 3 was higher in Chinese boys and girls across age.

**Table 2:** Length (cm)-for-age z-score curves at 0, ±1, ±2, and ± 3 SD for Chinese boys and girls from birth to 24 months

Age (month)	Boys										Girls									
	L	M	S	-3SD	-2SD	-1SD	0SD	1SD	2SD	3SD	L	M	S	-3SD	-2SD	-1SD	0SD	1SD	2SD	3SD
0	−0.7996	50.3	0.0306	46.0	47.4	48.8	50.3	51.9	53.6	55.3	−1.0973	49.9	0.0305	45.7	47.0	48.4	49.9	51.5	53.2	55.0
1	−0.7996	54.9	0.0318	50.1	51.6	53.2	54.9	56.7	58.6	60.6	−0.9519	54.2	0.0320	49.4	50.9	52.5	54.2	55.9	57.9	59.9
2	−0.7996	58.9	0.0326	53.6	55.3	57.1	58.9	60.9	63.0	65.3	−0.6922	57.9	0.0331	52.6	54.3	56.0	57.9	59.9	62.0	64.2
3	−0.7996	62.2	0.0330	56.6	58.3	60.2	62.2	64.3	66.6	69.0	−0.3981	61.0	0.0338	55.3	57.1	59.0	61.0	63.2	65.4	67.7
4	− 0.7996	64.8	0.0331	58.9	60.8	62.8	64.8	67.1	69.4	71.9	−0.1356	63.6	0.0342	57.4	59.4	61.4	63.6	65.8	68.1	70.5
5	−0.7996	67.0	0.0332	60.9	62.8	64.8	67.0	69.3	71.7	74.3	0.0640	65.7	0.0343	59.2	61.3	63.5	65.7	68.0	70.3	72.8
6	−0.7996	68.8	0.0331	62.6	64.5	66.6	68.8	71.2	73.7	76.3	0.2052	67.5	0.0344	60.8	63.0	65.2	67.5	69.9	72.3	74.8
7	−0.7996	70.5	0.0330	64.0	66.1	68.2	70.5	72.9	75.4	78.1	0.2974	69.1	0.0344	62.2	64.5	66.8	69.1	71.5	74.0	76.5
8	−0.7996	71.9	0.0330	65.4	67.4	69.6	71.9	74.4	76.9	79.7	0.3533	70.6	0.0344	63.5	65.8	68.2	70.6	73.0	75.6	78.1
9	−0.7996	73.2	0.0329	66.6	68.7	70.9	73.2	75.7	78.3	81.1	0.3867	71.9	0.0344	64.7	67.1	69.5	71.9	74.4	77.0	79.6
10	−0.7996	74.4	0.0329	67.7	69.8	72.0	74.4	76.9	79.6	82.5	0.4064	73.1	0.0343	65.8	68.2	70.7	73.1	75.7	78.3	80.9
11	−0.7996	75.5	0.0328	68.7	70.8	73.1	75.5	78.1	80.8	83.7	0.4167	74.3	0.0342	66.9	69.3	71.8	74.3	76.9	79.5	82.1
12	−0.7996	76.6	0.0328	69.7	71.9	74.2	76.6	79.2	82.0	84.9	0.4203	75.4	0.0342	67.9	70.3	72.8	75.4	78.0	80.6	83.3
13	−0.7996	77.7	0.0328	70.7	72.9	75.2	77.7	80.3	83.1	86.1	0.4205	76.5	0.0341	68.9	71.4	73.9	76.5	79.1	81.8	84.5
14	− 0.7996	78.8	0.0328	71.7	73.9	76.3	78.8	81.5	84.3	87.3	0.4189	77.6	0.0341	69.9	72.4	74.9	77.6	80.2	82.9	85.7
15	−0.7996	79.9	0.0327	72.7	74.9	77.3	79.9	82.6	85.4	88.5	0.4166	78.6	0.0340	70.9	73.4	76.0	78.6	81.3	84.1	86.9
16	−0.7996	81.0	0.0327	73.7	76.0	78.4	81.0	83.7	86.6	89.7	0.4142	79.7	0.0339	71.8	74.4	77.0	79.7	82.5	85.2	88.1
17	−0.7996	82.1	0.0327	74.7	77.0	79.4	82.1	84.8	87.8	90.9	0.4125	80.8	0.0339	72.8	75.4	78.1	80.8	83.6	86.4	89.2
18	−0.7996	83.1	0.0327	75.6	78.0	80.5	83.1	85.9	88.9	92.1	0.4121	81.8	0.0338	73.8	76.4	79.1	81.8	84.6	87.5	90.4
19	−0.7996	84.1	0.0327	76.6	78.9	81.5	84.1	87.0	90.0	93.2	0.4134	82.9	0.0337	74.7	77.4	80.1	82.9	85.7	88.6	91.5
20	−0.7996	85.2	0.0327	77.5	79.9	82.4	85.2	88.0	91.1	94.3	0.4154	83.9	0.0336	75.7	78.3	81.1	83.9	86.7	89.6	92.6
21	−0.7996	86.1	0.0327	78.4	80.8	83.4	86.1	89.0	92.1	95.4	0.4169	84.9	0.0336	76.6	79.3	82.0	84.9	87.7	90.7	93.7
22	−0.7996	87.1	0.0327	79.3	81.7	84.3	87.1	90.0	93.2	96.5	0.4172	85.8	0.0335	77.5	80.2	83.0	85.8	88.8	91.7	94.7
23	−0.7996	88.1	0.0327	80.1	82.6	85.3	88.1	91.0	94.2	97.5	0.4164	86.8	0.0335	78.4	81.1	83.9	86.8	89.8	92.7	95.8
24	−0.7996	89.0	0.0327	81.0	83.5	86.2	89.0	92.0	95.2	98.6	0.4146	87.8	0.0334	79.2	82.0	84.9	87.8	90.7	93.8	96.8

**Fig. 2:**
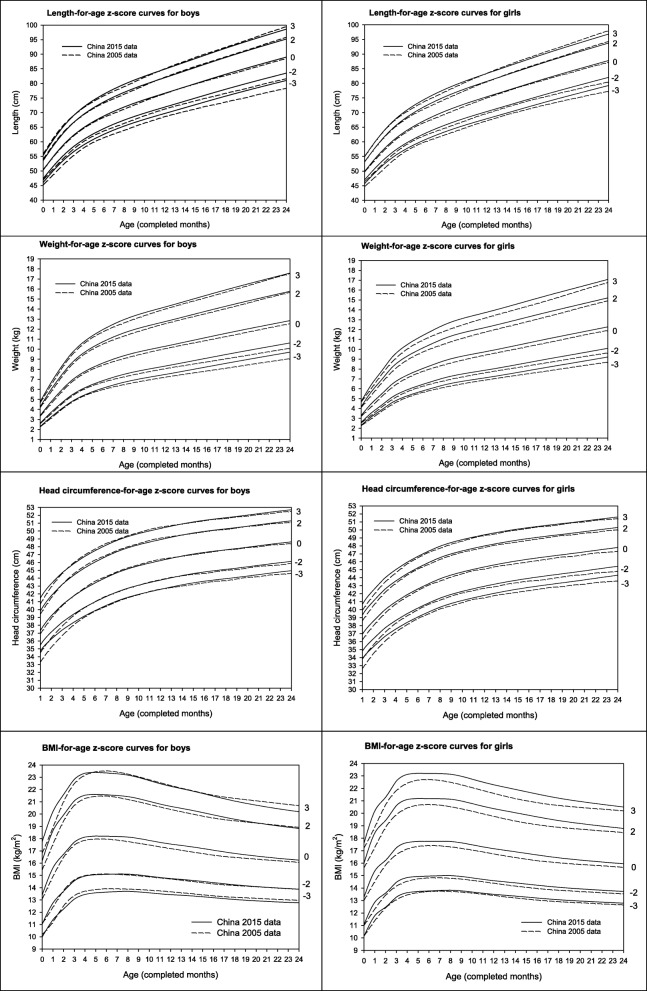
Comparison of growth-for-age z-score curves from China 2015 data (the present study) with those from China 2005 data in boys and girls

Compared to the China growth reference (2005 data), the median length-for-age in our study (2015 data) was on average 0.3 cm higher in boys, and 0.5 cm higher in girls across age (Fig. 2). This might be evidence of a small secular trend. The comparisons to the 2005 China references were more similar than that for the comparisons to the WHO standards (Figs. 1 and 2).

### Weight-for-age

Table 3 presents the growth reference of weight-for-age at 0, ±1, ±2, and ± 3 SD in our study. For weight-for-age z-score of − 2 (cutoff point for defining underweight), weight was on average 0.60 (range 0.13–0.94) kg heavier in Chinese boys and 0.80 (range 0.19–1.10) kg heavier in Chinese girls than those of WHO standards across age (Fig. 1).

**Table 3:** Weight (kg)-for-age z-score curves at 0, ±1, ±2, and ± 3 SD for Chinese boys and girls from birth to 24 months

Age (months)	Boys										Girls									
	L	M	S	-3SD	-2SD	-1SD	0SD	1SD	2SD	3SD	L	M	S	-3SD	-2SD	-1SD	0SD	1SD	2SD	3SD
0	0.3325	3.39	0.1213	2.30	2.63	2.99	3.39	3.82	4.28	4.78	0.0359	3.30	0.1196	2.30	2.59	2.92	3.30	3.71	4.18	4.71
1	0.3465	4.70	0.1196	3.21	3.66	4.16	4.70	5.29	5.92	6.60	−0.0184	4.51	0.1182	3.17	3.56	4.01	4.51	5.08	5.71	6.44
2	0.3315	5.87	0.1178	4.03	4.59	5.20	5.87	6.59	7.36	8.20	−0.0550	5.48	0.1168	3.88	4.35	4.88	5.48	6.17	6.94	7.81
3	0.2872	6.87	0.1159	4.76	5.41	6.11	6.87	7.70	8.60	9.57	−0.0886	6.46	0.1155	4.60	5.14	5.76	6.46	7.26	8.16	9.19
4	0.2373	7.61	0.1143	5.32	6.01	6.78	7.61	8.52	9.51	10.58	−0.1129	7.13	0.1145	5.09	5.69	6.37	7.13	8.01	9.00	10.13
5	0.1874	8.16	0.1130	5.75	6.48	7.28	8.16	9.13	10.18	11.33	−0.1331	7.63	0.1137	5.47	6.10	6.82	7.63	8.56	9.62	10.82
6	0.1414	8.61	0.1118	6.11	6.86	7.69	8.61	9.62	10.73	11.95	−0.1515	8.06	0.1130	5.79	6.45	7.20	8.06	9.03	10.14	11.41
7	0.1005	9.00	0.1107	6.42	7.19	8.05	9.00	10.05	11.20	12.48	−0.1690	8.45	0.1122	6.09	6.78	7.56	8.45	9.46	10.62	11.95
8	0.0648	9.34	0.1098	6.69	7.48	8.36	9.34	10.42	11.61	12.93	−0.1858	8.81	0.1115	6.37	7.08	7.89	8.81	9.86	11.07	12.45
9	0.0344	9.62	0.1089	6.92	7.73	8.62	9.62	10.72	11.95	13.31	−0.2003	9.11	0.1109	6.60	7.33	8.16	9.11	10.19	11.43	12.85
10	0.0082	9.86	0.1082	7.12	7.94	8.85	9.86	10.99	12.24	13.64	−0.2125	9.34	0.1103	6.78	7.53	8.38	9.34	10.44	11.71	13.17
11	−0.0159	10.08	0.1076	7.30	8.13	9.05	10.08	11.22	12.50	13.93	−0.2238	9.54	0.1098	6.94	7.70	8.56	9.54	10.67	11.95	13.44
12	−0.0398	10.29	0.1069	7.48	8.32	9.25	10.29	11.45	12.75	14.21	−0.2356	9.74	0.1093	7.10	7.87	8.74	9.74	10.88	12.19	13.70
13	−0.0650	10.51	0.1062	7.67	8.51	9.45	10.51	11.69	13.01	14.50	−0.2487	9.94	0.1088	7.26	8.04	8.93	9.94	11.10	12.43	13.97
14	−0.0919	10.73	0.1055	7.86	8.71	9.66	10.73	11.93	13.28	14.79	−0.2635	10.15	0.1082	7.44	8.23	9.13	10.15	11.33	12.69	14.26
15	−0.1199	10.95	0.1048	8.05	8.91	9.87	10.95	12.17	13.54	15.09	−0.2798	10.37	0.1076	7.61	8.41	9.33	10.37	11.57	12.95	14.55
16	−0.1485	11.18	0.1040	8.24	9.11	10.08	11.18	12.41	13.81	15.39	−0.2974	10.59	0.1070	7.80	8.61	9.53	10.59	11.81	13.21	14.84
17	−0.1776	11.40	0.1033	8.43	9.30	10.29	11.40	12.65	14.07	15.68	−0.3161	10.81	0.1063	7.98	8.80	9.74	10.81	12.05	13.47	15.13
18	−0.2070	11.62	0.1026	8.62	9.50	10.49	11.62	12.88	14.32	15.96	−0.3357	11.03	0.1057	8.16	8.99	9.94	11.03	12.28	13.73	15.42
19	−0.2365	11.83	0.1019	8.80	9.69	10.70	11.83	13.11	14.58	16.25	−0.3558	11.24	0.1051	8.34	9.18	10.14	11.24	12.51	13.98	15.70
20	−0.2659	12.04	0.1012	8.99	9.88	10.89	12.04	13.34	14.82	16.52	−0.3766	11.45	0.1045	8.51	9.36	10.33	11.45	12.73	14.23	15.98
21	−0.2953	12.24	0.1006	9.17	10.07	11.09	12.24	13.56	15.06	16.79	−0.3981	11.65	0.1039	8.69	9.54	10.53	11.65	12.96	14.48	16.25
22	−0.3246	12.45	0.0999	9.35	10.26	11.28	12.45	13.78	15.30	17.06	−0.4204	11.86	0.1033	8.86	9.73	10.72	11.86	13.18	14.72	16.53
23	−0.3539	12.65	0.0993	9.53	10.44	11.47	12.65	13.99	15.54	17.33	−0.4432	12.07	0.1027	9.04	9.91	10.92	12.07	13.41	14.97	16.81
24	−0.3832	12.85	0.0986	9.71	10.62	11.66	12.85	14.21	15.77	17.59	−0.4665	12.28	0.1021	9.22	10.10	11.11	12.28	13.63	15.21	17.08

Compared to China reference from 2005 data, the weight-for-age median in our study (China 2015 data) was on average 0.25 kg higher (range 0.07–0.33 kg) in boys, and 0.34 kg higher (range 0.09–0.42 kg) in girls across age (Fig. 2).

### Head circumference-for-age

Table 4 presents the growth reference of head circumference-for-age at 0, ±1, ±2, and ± 3 SD in our study. At the z-score of − 2, head circumference was 0.36 cm greater (range 0.08 to 0.86 cm) in Chinese boys, and 0.76 cm greater (range 0.54 to 1.04 cm) in Chinese girls, than the corresponding WHO standards (Fig. 1).

**Table 4:** Head circumference (cm)-for-age z-score curves at 0, ±1, ±2, and ± 3 SD for Chinese boys and girls from birth to 24 months

Age (months)	Boys										Girls									
	L	M	S	-3SD	-2SD	-1SD	0SD	1SD	2SD	3SD	L	M	S	-3SD	-2SD	-1SD	0SD	1SD	2SD	3SD
0	−7.0263	34.3	0.0262	32.2	32.8	33.4	34.3	35.3	36.6	38.4	−1.4337	34.1	0.0300	31.3	32.1	33.1	34.1	35.1	36.3	37.5
1	−4.4686	37.3	0.0280	34.8	35.5	36.4	37.3	38.5	39.8	41.5	−1.2431	36.9	0.0304	33.9	34.8	35.8	36.9	38.1	39.3	40.7
2	−2.7515	39.2	0.0291	36.3	37.1	38.1	39.2	40.4	41.8	43.3	−1.0791	38.6	0.0304	35.4	36.4	37.4	38.6	39.8	41.1	42.5
3	−1.7763	40.6	0.0294	37.4	38.4	39.5	40.6	41.8	43.2	44.7	−0.9472	40.0	0.0303	36.6	37.7	38.8	40.0	41.2	42.5	43.9
4	−1.2600	41.7	0.0295	38.3	39.4	40.5	41.7	43.0	44.3	45.8	−0.8444	41.0	0.0301	37.6	38.6	39.8	41.0	42.2	43.6	45.0
5	−0.9752	42.6	0.0293	39.2	40.2	41.4	42.6	43.9	45.3	46.7	−0.7629	41.8	0.0298	38.4	39.5	40.6	41.8	43.1	44.5	45.9
6	−0.7968	43.4	0.0291	39.9	41.0	42.1	43.4	44.7	46.0	47.5	−0.6960	42.6	0.0295	39.1	40.2	41.4	42.6	43.9	45.2	46.7
7	−0.6658	44.1	0.0289	40.5	41.6	42.8	44.1	45.4	46.7	48.2	−0.6377	43.3	0.0292	39.7	40.9	42.0	43.3	44.6	45.9	47.4
8	−0.5616	44.7	0.0286	41.1	42.2	43.4	44.7	46.0	47.4	48.8	−0.5863	43.9	0.0289	40.3	41.5	42.6	43.9	45.2	46.5	48.0
9	−0.4796	45.2	0.0284	41.6	42.7	43.9	45.2	46.5	47.9	49.3	−0.5444	44.3	0.0286	40.8	41.9	43.1	44.3	45.6	47.0	48.4
10	−0.4171	45.6	0.0282	41.9	43.1	44.3	45.6	46.9	48.2	49.7	−0.5097	44.7	0.0283	41.2	42.3	43.5	44.7	46.0	47.4	48.8
11	−0.3699	45.9	0.0280	42.3	43.4	44.7	45.9	47.2	48.6	50.0	−0.4793	45.1	0.0280	41.5	42.6	43.8	45.1	46.3	47.7	49.1
12	−0.3349	46.2	0.0278	42.6	43.8	45.0	46.2	47.5	48.9	50.3	−0.4516	45.4	0.0278	41.8	42.9	44.1	45.4	46.6	48.0	49.4
13	−0.3116	46.5	0.0277	42.9	44.1	45.3	46.5	47.8	49.2	50.6	−0.4262	45.6	0.0275	42.1	43.2	44.4	45.6	46.9	48.3	49.6
14	−0.3002	46.8	0.0275	43.2	44.3	45.6	46.8	48.1	49.5	50.9	−0.4029	45.9	0.0273	42.3	43.5	44.7	45.9	47.2	48.5	49.9
15	−0.2977	47.1	0.0274	43.4	44.6	45.8	47.1	48.4	49.7	51.1	− 0.3819	46.1	0.0271	42.6	43.7	44.9	46.1	47.4	48.7	50.1
16	−0.2997	47.3	0.0273	43.6	44.8	46.0	47.3	48.6	49.9	51.4	−0.3629	46.3	0.0269	42.8	43.9	45.1	46.3	47.6	48.9	50.3
17	−0.3039	47.4	0.0272	43.8	45.0	46.2	47.4	48.8	50.1	51.5	−0.3456	46.5	0.0267	43.0	44.1	45.3	46.5	47.8	49.1	50.5
18	−0.3091	47.6	0.0271	43.9	45.1	46.3	47.6	48.9	50.3	51.7	−0.3293	46.7	0.0265	43.2	44.3	45.5	46.7	48.0	49.3	50.6
19	−0.3155	47.8	0.0270	44.1	45.3	46.5	47.8	49.1	50.4	51.9	−0.3135	46.9	0.0263	43.4	44.5	45.7	46.9	48.1	49.4	50.8
20	− 0.3232	47.9	0.0269	44.3	45.5	46.7	47.9	49.3	50.6	52.0	−0.2976	47.1	0.0261	43.6	44.7	45.9	47.1	48.3	49.6	50.9
21	−0.3320	48.1	0.0268	44.5	45.6	46.9	48.1	49.4	50.8	52.2	−0.2811	47.2	0.0260	43.7	44.9	46.0	47.2	48.5	49.8	51.1
22	−0.3414	48.3	0.0267	44.6	45.8	47.0	48.3	49.6	51.0	52.4	−0.2641	47.4	0.0258	43.9	45.1	46.2	47.4	48.7	50.0	51.3
23	−0.3506	48.5	0.0266	44.8	46.0	47.2	48.5	49.8	51.1	52.6	−0.2473	47.6	0.0256	44.1	45.3	46.4	47.6	48.9	50.1	51.5
24	−0.3592	48.6	0.0265	45.0	46.1	47.4	48.6	49.9	51.3	52.7	−0.2313	47.8	0.0254	44.3	45.4	46.6	47.8	49.0	50.3	51.6

Compared to cross-sectional 2005 norms for China, the median head circumference-for-age in our study was similar in boys, but on average 0.3 cm greater (range 0.1–0.7 cm) in girls across age (Fig. 2).

### BMI-for-age

Table 5 presents the growth reference of BMI-for-age at 0, ±1, ±2, and ± 3 SD in our study. As shown in Fig. 1, median BMI-for-age was on average 0.70 kg/m^2^ (range 0.01 to 0.92 kg/m^2^) higher in Chinese boys, and 0.7 (range 0.0 to 1.0) kg/m^2^ higher in Chinese girls than the corresponding WHO standards across the age of 0–24 months. For z-score of 2, BMI on average ~ 0.70 kg/m^2^ higher in Chinese boys and girls than the WHO standards.

**Table 5:** BMI-for-age z-score at 0, ±1, ±2, and ± 3 SD for Chinese boys and girls from birth to 24 months

Age (month)	Boys										Girls									
	L	M	S	-3SD	-2SD	-1SD	0SD	1SD	2SD	3SD	L	M	S	-3SD	-2SD	-1SD	0SD	1SD	2SD	3SD
0	0.1590	13.4	0.0958	10.0	11.0	12.2	13.4	14.7	16.2	17.8	−0.1727	13.3	0.0920	10.1	11.1	12.1	13.3	14.5	16.0	17.6
1	0.7178	15.6	0.0943	11.4	12.8	14.2	15.6	17.1	18.6	20.2	−0.0900	15.4	0.0898	11.8	12.9	14.1	15.4	16.8	18.4	20.2
2	0.6937	16.7	0.0930	12.3	13.7	15.2	16.7	18.3	19.9	21.6	−0.1078	16.2	0.0891	12.5	13.6	14.8	16.2	17.7	19.4	21.3
3	0.6483	17.7	0.0920	13.1	14.6	16.1	17.7	19.4	21.1	22.8	−0.1322	17.2	0.0886	13.3	14.5	15.8	17.2	18.8	20.6	22.6
4	0.6054	18.1	0.0909	13.5	14.9	16.5	18.1	19.8	21.5	23.3	−0.1567	17.7	0.0880	13.6	14.8	16.2	17.7	19.3	21.1	23.1
5	0.5683	18.2	0.0899	13.6	15.1	16.6	18.2	19.9	21.6	23.4	−0.1810	17.7	0.0875	13.7	14.9	16.3	17.7	19.4	21.2	23.2
6	0.5384	18.2	0.0890	13.6	15.1	16.6	18.2	19.8	21.6	23.4	−0.2055	17.7	0.0869	13.8	15.0	16.3	17.7	19.4	21.2	23.2
7	0.5161	18.2	0.0880	13.7	15.1	16.6	18.2	19.8	21.5	23.3	−0.2298	17.8	0.0863	13.8	15.0	16.3	17.8	19.4	21.2	23.2
8	0.5000	18.1	0.0871	13.7	15.1	16.6	18.1	19.7	21.4	23.2	−0.2535	17.7	0.0857	13.8	15.0	16.3	17.7	19.3	21.1	23.1
9	0.4888	18.0	0.0862	13.7	15.0	16.5	18.0	19.6	21.3	23.0	−0.2763	17.6	0.0851	13.8	14.9	16.2	17.6	19.2	21.0	23.0
10	0.4814	17.9	0.0854	13.6	14.9	16.4	17.9	19.4	21.0	22.7	−0.2981	17.5	0.0845	13.7	14.8	16.1	17.5	19.0	20.8	22.7
11	0.4767	17.7	0.0846	13.5	14.8	16.2	17.7	19.2	20.8	22.5	−0.3192	17.3	0.0839	13.6	14.7	15.9	17.3	18.8	20.6	22.5
12	0.4735	17.6	0.0838	13.4	14.7	16.1	17.6	19.1	20.6	22.3	−0.3398	17.2	0.0834	13.5	14.6	15.8	17.2	18.7	20.4	22.3
13	0.4713	17.4	0.0830	13.4	14.7	16.0	17.4	18.9	20.5	22.1	−0.3598	17.0	0.0828	13.4	14.5	15.7	17.0	18.5	20.2	22.1
14	0.4693	17.3	0.0823	13.3	14.6	15.9	17.3	18.8	20.3	21.9	−0.3794	16.9	0.0823	13.4	14.4	15.6	16.9	18.4	20.0	21.9
15	0.4673	17.2	0.0816	13.3	14.5	15.8	17.2	18.6	20.1	21.7	−0.3984	16.8	0.0818	13.3	14.3	15.5	16.8	18.2	19.9	21.7
16	0.4654	17.1	0.0809	13.2	14.4	15.7	17.1	18.5	19.9	21.5	−0.4169	16.7	0.0814	13.2	14.2	15.4	16.7	18.1	19.7	21.5
17	0.4635	16.9	0.0802	13.1	14.3	15.6	16.9	18.3	19.7	21.3	−0.4349	16.5	0.0809	13.1	14.1	15.3	16.5	18.0	19.6	21.4
18	0.4616	16.8	0.0795	13.0	14.2	15.5	16.8	18.2	19.6	21.1	−0.4524	16.4	0.0805	13.1	14.1	15.2	16.4	17.8	19.4	21.2
19	0.4598	16.7	0.0789	13.0	14.2	15.4	16.7	18.0	19.4	20.9	−0.4695	16.3	0.0801	13.0	14.0	15.1	16.3	17.7	19.3	21.1
20	0.4581	16.6	0.0783	12.9	14.1	15.3	16.6	17.9	19.3	20.7	−0.4862	16.2	0.0797	13.0	13.9	15.0	16.2	17.6	19.2	20.9
21	0.4564	16.5	0.0777	12.9	14.0	15.2	16.5	17.8	19.2	20.6	−0.5024	16.2	0.0793	12.9	13.9	15.0	16.2	17.5	19.1	20.8
22	0.4547	16.4	0.0772	12.8	14.0	15.2	16.4	17.7	19.0	20.4	−0.5181	16.1	0.0789	12.9	13.8	14.9	16.1	17.4	19.0	20.7
23	0.4531	16.3	0.0766	12.8	13.9	15.1	16.3	17.6	18.9	20.3	−0.5334	16.0	0.0786	12.8	13.8	14.8	16.0	17.4	18.9	20.6
24	0.4516	16.2	0.0761	12.8	13.9	15.0	16.2	17.5	18.8	20.2	−0.5482	16.0	0.0782	12.8	13.7	14.8	16.0	17.3	18.8	20.5

Compared to the China corresponding growth references from 2005 data, the median BMI-for-age in our study was on average 0.3 kg/m^2^ higher in boys and 0.4 kg/m^2^ higher in girls across age (Fig. 2).

### Weight-for-length

**Fig. 3:**
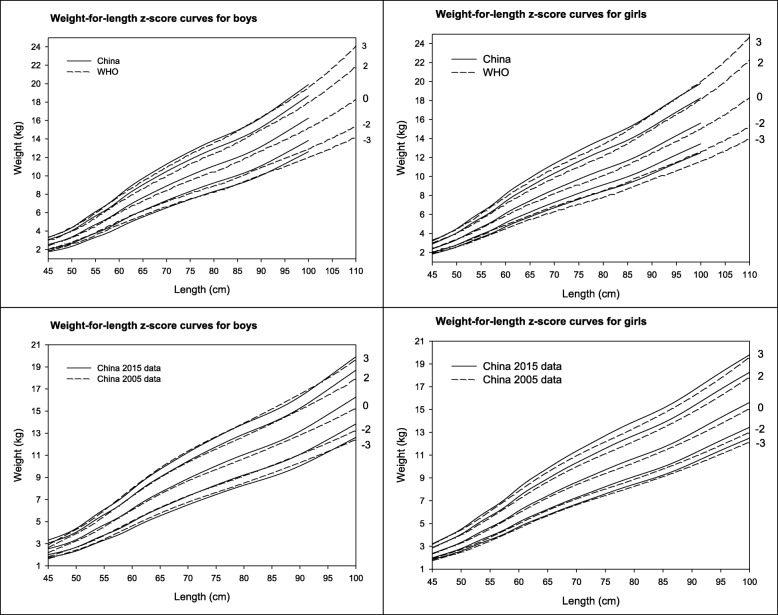
Comparison of weight-for-length z-score curves from China 2015 data with the WHO standards and Chinese references from 2005 data in boys and girls

Table 6 presents the growth references of weight-for-length at 0, ±1, ±2, and ± 3 SD in our study. Median weight-for-length was on average 0.43 kg greater (range 0.01 to 1.07 kg) than WHO standards in boys, and 0.42 kg greater (range 0.00 to 0.64 kg) in Chinese girls from body length > 50 cm (Fig. 3), but lighter weight at the very short length in Chinese girls (< 52 cm).

**Table 6:** Weight (kg)- for-length (cm) z-score curves at 0, ±1, ±2, and ± 3 SD for Chinese boys and girls from birth to 24 months

Length (cm)	Boys										Girls									
	L	M	S	-3SD	-2SD	-1SD	0SD	1SD	2SD	3SD	L	M	S	-3SD	-2SD	-1SD	0SD	1SD	2SD	3SD
45	1.0000	2.54	0.1032	1.76	2.02	2.28	2.54	2.80	3.07	3.33	−0.5434	2.37	0.0928	1.83	1.99	2.17	2.37	2.61	2.89	3.21
46	1.0000	2.67	0.1028	1.85	2.12	2.40	2.67	2.95	3.22	3.50	−0.5303	2.56	0.0927	1.97	2.14	2.34	2.56	2.81	3.11	3.46
47	1.0000	2.81	0.1025	1.95	2.24	2.53	2.81	3.10	3.39	3.68	−0.5173	2.74	0.0927	2.12	2.30	2.51	2.74	3.02	3.33	3.70
48	1.0000	2.97	0.1021	2.06	2.36	2.66	2.97	3.27	3.57	3.88	−0.5041	2.93	0.0926	2.26	2.45	2.68	2.93	3.22	3.56	3.95
49	1.0000	3.15	0.1017	2.19	2.51	2.83	3.15	3.47	3.79	4.11	−0.4904	3.12	0.0926	2.41	2.62	2.85	3.12	3.44	3.79	4.21
50	1.0000	3.36	0.1012	2.34	2.68	3.02	3.36	3.70	4.04	4.38	−0.4757	3.34	0.0925	2.58	2.80	3.06	3.34	3.68	4.06	4.50
51	1.0000	3.61	0.1007	2.52	2.88	3.25	3.61	3.97	4.34	4.70	−0.4597	3.60	0.0924	2.77	3.02	3.29	3.60	3.96	4.37	4.84
52	1.0000	3.89	0.1001	2.72	3.11	3.50	3.89	4.28	4.67	5.06	−0.4434	3.88	0.0923	2.99	3.25	3.55	3.88	4.26	4.71	5.22
53	1.0000	4.18	0.0995	2.93	3.35	3.76	4.18	4.59	5.01	5.42	−0.4276	4.16	0.0922	3.20	3.48	3.80	4.16	4.57	5.04	5.58
54	1.0000	4.45	0.0990	3.13	3.57	4.01	4.45	4.89	5.33	5.77	−0.4126	4.42	0.0921	3.40	3.70	4.03	4.42	4.85	5.35	5.92
55	1.0000	4.71	0.0984	3.32	3.78	4.25	4.71	5.17	5.64	6.10	−0.3983	4.66	0.0920	3.58	3.90	4.26	4.66	5.12	5.64	6.24
56	1.0000	4.95	0.0978	3.50	3.99	4.47	4.95	5.44	5.92	6.41	−0.3841	4.90	0.0918	3.77	4.10	4.48	4.90	5.38	5.93	6.56
57	1.0000	5.21	0.0972	3.69	4.20	4.70	5.21	5.72	6.22	6.73	−0.3702	5.16	0.0917	3.98	4.33	4.72	5.16	5.67	6.24	6.90
58	1.0000	5.49	0.0966	3.90	4.43	4.96	5.49	6.02	6.55	7.08	−0.3572	5.46	0.0915	4.21	4.58	4.99	5.46	6.00	6.60	7.29
59	1.0000	5.81	0.0959	4.14	4.69	5.25	5.81	6.36	6.92	7.48	−0.3463	5.79	0.0912	4.46	4.85	5.29	5.79	6.35	6.99	7.72
60	1.0000	6.14	0.0952	4.39	4.97	5.56	6.14	6.73	7.31	7.90	−0.3376	6.11	0.0910	4.71	5.12	5.59	6.11	6.70	7.38	8.14
61	1.0000	6.48	0.0945	4.64	5.25	5.87	6.48	7.09	7.70	8.32	−0.3307	6.41	0.0907	4.94	5.38	5.86	6.41	7.03	7.73	8.53
62	1.0000	6.79	0.0938	4.88	5.52	6.16	6.79	7.43	8.07	8.71	−0.3252	6.69	0.0904	5.16	5.61	6.12	6.69	7.33	8.06	8.88
63	1.0000	7.09	0.0931	5.11	5.77	6.43	7.09	7.75	8.41	9.07	−0.3212	6.94	0.0901	5.35	5.82	6.35	6.94	7.60	8.35	9.21
64	1.0000	7.36	0.0925	5.32	6.00	6.68	7.36	8.05	8.73	9.41	−0.3184	7.19	0.0898	5.55	6.04	6.58	7.19	7.87	8.65	9.52
65	1.0000	7.63	0.0918	5.53	6.23	6.93	7.63	8.33	9.03	9.73	−0.3169	7.44	0.0894	5.75	6.25	6.81	7.44	8.14	8.94	9.84
66	1.0000	7.89	0.0912	5.73	6.45	7.17	7.89	8.61	9.33	10.05	−0.3170	7.69	0.0891	5.95	6.47	7.04	7.69	8.42	9.24	10.17
67	1.0000	8.14	0.0906	5.93	6.67	7.40	8.14	8.88	9.62	10.36	−0.3188	7.94	0.0887	6.15	6.68	7.27	7.94	8.68	9.53	10.48
68	1.0000	8.39	0.0900	6.13	6.88	7.64	8.39	9.15	9.90	10.66	−0.3223	8.18	0.0883	6.34	6.89	7.49	8.18	8.94	9.81	10.78
69	1.0000	8.64	0.0894	6.32	7.10	7.87	8.64	9.42	10.19	10.96	−0.3270	8.41	0.0879	6.53	7.09	7.71	8.41	9.19	10.08	11.08
70	1.0000	8.89	0.0889	6.52	7.31	8.10	8.89	9.68	10.47	11.26	−0.3328	8.63	0.0875	6.71	7.28	7.92	8.63	9.43	10.34	11.36
71	1.0000	9.13	0.0883	6.71	7.52	8.33	9.13	9.94	10.74	11.55	−0.3391	8.85	0.0871	6.89	7.47	8.12	8.85	9.67	10.59	11.63
72	1.0000	9.36	0.0878	6.90	7.72	8.54	9.36	10.19	11.01	11.83	−0.3458	9.06	0.0867	7.07	7.66	8.32	9.06	9.90	10.84	11.90
73	1.0000	9.59	0.0872	7.08	7.92	8.76	9.59	10.43	11.27	12.10	−0.3527	9.28	0.0863	7.25	7.85	8.53	9.28	10.13	11.09	12.18
74	1.0000	9.82	0.0867	7.26	8.12	8.97	9.82	10.67	11.52	12.37	−0.3597	9.50	0.0858	7.43	8.04	8.73	9.50	10.37	11.34	12.45
75	1.0000	10.04	0.0861	7.44	8.31	9.17	10.04	10.90	11.77	12.63	−0.3667	9.72	0.0854	7.61	8.23	8.93	9.72	10.60	11.59	12.72
76	1.0000	10.26	0.0856	7.62	8.50	9.38	10.26	11.13	12.01	12.89	−0.3737	9.93	0.0850	7.78	8.42	9.13	9.93	10.83	11.84	12.98
77	1.0000	10.47	0.0851	7.80	8.69	9.58	10.47	11.36	12.25	13.14	−0.3806	10.14	0.0846	7.96	8.60	9.33	10.14	11.05	12.07	13.24
78	1.0000	10.68	0.0846	7.97	8.87	9.78	10.68	11.58	12.49	13.39	−0.3872	10.34	0.0842	8.13	8.78	9.52	10.34	11.26	12.30	13.48
79	1.0000	10.88	0.0841	8.14	9.05	9.97	10.88	11.80	12.71	13.63	−0.3933	10.53	0.0837	8.29	8.96	9.70	10.53	11.47	12.53	13.72
80	1.0000	11.07	0.0836	8.30	9.22	10.15	11.07	12.00	12.93	13.85	−0.3988	10.72	0.0834	8.45	9.12	9.88	10.72	11.67	12.74	13.95
81	1.0000	11.26	0.0832	8.45	9.39	10.32	11.26	12.20	13.13	14.07	−0.4036	10.91	0.0830	8.60	9.29	10.05	10.91	11.87	12.95	14.18
82	1.0000	11.44	0.0828	8.60	9.55	10.49	11.44	12.39	13.33	14.28	−0.4076	11.09	0.0826	8.76	9.45	10.22	11.09	12.06	13.16	14.40
83	1.0000	11.62	0.0823	8.75	9.71	10.67	11.62	12.58	13.54	14.49	−0.4109	11.28	0.0822	8.92	9.62	10.40	11.28	12.26	13.37	14.63
84	1.0000	11.81	0.0819	8.91	9.87	10.84	11.81	12.78	13.74	14.71	−0.4134	11.47	0.0819	9.08	9.79	10.58	11.47	12.47	13.59	14.86
85	1.0000	12.00	0.0815	9.07	10.05	11.03	12.00	12.98	13.96	14.94	−0.4149	11.68	0.0815	9.25	9.98	10.78	11.68	12.69	13.83	15.11
86	1.0000	12.21	0.0810	9.24	10.23	11.22	12.21	13.20	14.19	15.18	−0.4151	11.90	0.0811	9.44	10.17	10.99	11.90	12.92	14.08	15.38
87	1.0000	12.43	0.0806	9.42	10.42	11.42	12.43	13.43	14.43	15.43	−0.4137	12.13	0.0807	9.63	10.38	11.21	12.13	13.17	14.34	15.66
88	1.0000	12.66	0.0801	9.62	10.63	11.64	12.66	13.67	14.69	15.70	−0.4105	12.38	0.0803	9.84	10.60	11.44	12.38	13.44	14.62	15.96
89	1.0000	12.91	0.0797	9.82	10.85	11.88	12.91	13.94	14.96	15.99	−0.4056	12.65	0.0799	10.06	10.83	11.69	12.65	13.72	14.92	16.28
90	1.0000	13.17	0.0792	10.04	11.09	12.13	13.17	14.22	15.26	16.30	−0.3987	12.92	0.0796	10.28	11.07	11.94	12.92	14.01	15.23	16.60
91	1.0000	13.46	0.0787	10.28	11.34	12.40	13.46	14.52	15.58	16.64	−0.3901	13.19	0.0792	10.51	11.31	12.20	13.19	14.30	15.54	16.93
92	1.0000	13.75	0.0782	10.53	11.60	12.68	13.75	14.83	15.91	16.98	−0.3797	13.47	0.0789	10.74	11.56	12.47	13.47	14.60	15.86	17.27
93	1.0000	14.06	0.0778	10.78	11.87	12.97	14.06	15.15	16.25	17.34	−0.3677	13.75	0.0786	10.97	11.80	12.73	13.75	14.89	16.17	17.60
94	1.0000	14.37	0.0773	11.04	12.15	13.26	14.37	15.48	16.59	17.71	−0.3543	14.03	0.0783	11.20	12.05	12.99	14.03	15.19	16.48	17.93
95	1.0000	14.68	0.0769	11.30	12.43	13.56	14.68	15.81	16.94	18.07	−0.3398	14.30	0.0780	11.42	12.29	13.24	14.30	15.48	16.79	18.25
96	1.0000	15.00	0.0764	11.56	12.71	13.85	15.00	16.15	17.29	18.44	−0.3243	14.57	0.0777	11.64	12.52	13.50	14.57	15.77	17.10	18.57
97	1.0000	15.32	0.0760	11.82	12.99	14.15	15.32	16.48	17.65	18.81	−0.3081	14.84	0.0775	11.86	12.76	13.75	14.84	16.05	17.39	18.89
98	1.0000	15.63	0.0756	12.09	13.27	14.45	15.63	16.82	18.00	19.18	−0.2914	15.10	0.0772	12.07	12.99	13.99	15.10	16.33	17.69	19.20
99	1.0000	15.95	0.0751	12.36	13.56	14.75	15.95	17.15	18.35	19.55	−0.2744	15.37	0.0769	12.29	13.22	14.24	15.37	16.61	17.98	19.51
100	1.0000	16.27	0.0747	12.62	13.84	15.06	16.27	17.49	18.70	19.92	−0.2575	15.63	0.0767	12.50	13.45	14.49	15.63	16.89	18.28	19.81

For z-score of − 2 (cutoff for wasting definition) in boys, weight was ~ 0.29 kg higher (range 0.003–0.94 kg) than the WHO standard at length > 64 cm; between length 45–63 cm, it was 0.08 kg lower (ranged 0.02 to − 0.17) (Fig. 3). In Chinese girls, the weight-for-length values at z-score of − 2 were on average 0.44 kg heavier (ranging 0.001 to 0.85 kg) than the WHO standards for length > 49 cm. For z-score of 2 (cutoff for overweight definition), compared to the WHO standards, weight was on average 0.39 kg higher (range 0.04 to 0.75 kg) in Chinese boys, and 0.34 kg higher (range 0.06 to 0.64 kg) in Chinese girls for the length > 50 cm. Similarly, for z-score of 3, weight-for-length was on average 0.16 kg higher (range − 0.11to 0.36 kg) in Chinese boys, and was 0.30 kg higher (range 0.00 to 0.64 kg) at most length (49 cm to 95 cm) in Chinese girls than the WHO standards.

Compared to cross-sectional 2005 growth references for China, the median weight-for-length was on average 0.31 kg-cm higher (range 0.03–1.00 kg-cm) in boys and 0.28 (range 0.02–0.56) kg-cm higher in girls across length in this study (Fig. 3).

### The difference between our raw data and WHO standards

The numbers of anthropometric measurements used for generating smoothed growth curves was shown in Additional file 1: Tables S2 and S3. This study measured the children at 7 targeted ages (42 days, 3, 6, 9, 12, 18 and 24 months), but in fact provided adequate monthly numbers in the first 12 months (Additional file 1: Tables S2 and S3). In addition to above comparison of the LMS-method-fitted smoothing curves, we also presented the 3rd, 10th, 50th, 90th and 97th percentiles of growth measures by age in both boys (Additional file 1: Table S4) and girls (Additional file 1: Table S5). Compared to the corresponding 2006 WHO percentile standards, the 3rd, 10th, 50th, 90th and 97th percentiles (across the ages evaluated in this study from 0 to 2 years) for length, weight, and BMI (Additional file 1: Table S4 for boys and Additional file 1: Table S5 for girls) were consistently higher in healthy Chinese boys (Additional file 1: Table S6) and girls (Additional file 1: Table S7) in 2015. For example, the median lengths from 0 to 2 years were 50.0–89.5 cm in boys (Additional file 1: Table S4), which were 0.1–3.1 cm taller than the WHO percentile standards (Additional file 1: Table S6). The differences compared to WHO standards also were present for weight by length in both boys and girls (Additional file 1: Tables S8 and S9). This indicates the robust of our results.

## Discussion

This report of growth measures is based on a large cohort of children (*n* = 4251) from six recent birth cohorts from China. Growth references from this study represent normal growth of today’s Chinese children from birth to 24 months by using the multicenter data collected recently (from 2012 to 2015). Compared with the WHO standards (collected more than 10 years ago from mid-1997 to end of 2003) and the current China references (collected 10-years ago in late 2005), the median values of length-, weight-, and BMI-for-age reported here were all higher across the ages from 0 to 2 years, and also for median head circumference-for-age except for boys in our study compared to the 2005 references for China. The weight-for-length in our study was also slightly higher at most times in both boys and girls. The magnitude of differences between the WHO standards and the current large cohort (assessed in 2015) was larger than the magnitude of differences previously reported compared to the outdated 2005 references for China. Our report provides improved references for evaluating growth of children aged 0–24 months in modern China.

The height- and weight-for-age values were higher in our longitudinal cohort assessed in five cities of China (Shanghai, Ma’anshan Anhui, Wuhan, Jiangsu, and Guangzhou) than in the cohort based on a cross-sectional study in nine cities of China (Beijing, Shanghai, Harbin, Xi’an, Nanjing, Wuhan, Guangzhou, Fuzhou, and Kunming) [3]. This could be a secular trend. The CBCC cohorts recruited pregnant women in provincial or large tertiary maternity and child hospitals. Most mothers had high education (college or higher), maternal smoking was rare, and the living standard were relatively high. Thus, the growth data in this study may reflect infant growth patterns under near-optimal circumstances. Since our data were acquired recently (10 years since 2005), the higher length and weight may also reflect an ongoing secular trend [4]. The WHO data suggest that secular trend may depend on where the cohort was acquired: the predicted adult height from the child’s length at 2 years suggested there would be no parent-offspring difference in Norway and the United States (i.e., no increase due to a secular trend), but the predicted adult height was much larger than mid-parental height for the other four countries (Brazil, Ghana, India and Oman). [15] Based on the taller height reported here for ages 0 to 24 months than the 2005 China data, we expect a secular trend (i.e., we predict that average adulthood the height of the children in China will exceed the average height of their parents). While China has undergone dramatic progress in economic and social development, the differences still exist between urban and rural areas, different ethnics, and different social economic. The growth pattern observed in this study may reflect infant growth patterns under more optimal circumstances.

Some studies have found that some child population might have their own growth pattern [16], and our study confirmed that Chinese children may be one of them [3, 17, 18]. The difference in values for height-, weight- and BMI-for-age, weight-for-length, and head circumference in this report in comparison to the WHO standards suggests an interesting country difference, and adds to previous comparison that have been summarized in a recent review [19]. Based on studies from both longitudinal and cross-sectional designs, this review concluded that the WHO standards for height and weight “… endorsed slenderness in the midst of an obesity epidemic” and for head circumference were underestimates (and “… would put many children at risk for misdiagnosis of macrocephaly and microcephaly”). Healthy children in some countries are classified (perhaps inappropriately) as “stunted” [16]. In opposite of findings from some countries (overestimating stunting) [16], overall, our study confirmed that the values of growth measures were higher for the key z-score cutoffs in Chinese children in comparison with WHO growth standards [3, 5].

Our references provide the potential cutoffs for evaluating child growth in a population (like in modern China), where children are the center of attention in the family and are growing under favorable environments. Length has been widely used in early detection of stunting, while weight is commonly used as a measure responsive to short-term influences [20]. Head circumference is then the next most-used measure in clinical settings. To reflect the growth centile (position) of a Chinese child in local population, conditioned on age and sex, the Chinese growth standards need to be considered. It may help identify the infants who suffer from poor and modifiable conditions, and thus target those who may benefit most from intervention. In this study, while another term was considered (“growth pattern”), the term “growth reference” was used to maintain consistency with the term used in other publications about Chinese cohorts and to contrast to the term “growth standard” used for the WHO cohort.

One characteristic of this study (the large-scale multicenter prospective birth cohort design) allows us to obtain data on pre- and perinatal risk factors including GDM, chronic hypertension, pre-eclampsia and preterm status. Based on this strength, we could exclude affected mother-infant pairs cases at risk for abnormal patterns of child growth. In this study, the difference of mean paternal age among the three groups of children (mothers with GDM, born preterm, and healthy children) is interesting. Older fathers have more de novo mutations in DNA, and this probably contributes to growth in some cases [21]. Another strength of this study is the longitudinal rather than cross-sectional design. Additional longitudinal analysis [22, 23] of these longitudinal data could better capture and describe the tempo of growth, but due to space limitations will be presented elsewhere. Also, in this sample the educational level of mothers was high, and few of the mothers smoked, so the children lived in advantaged condition, and approach the criteria used for establishing the WHO standards (reflecting how children should grow). Therefore, the data here may reflect growth in near-optimal conditions in China, and provide a growth pattern for contemporary Chinese children.

On the other hand, one limitation of this study is that in some cases head circumference at birth was not measured, and some of children were just followed up to 12 months, which reduced the sample size for this measurement. However, our sample size is still larger than the sample sizes in similar longitudinal birth cohort studies conducted in other countries. We have also performed sensitivity analysis to summary the 3rd, 10th, 50th, 90th and 97th percentiles of all growth measures in infant who had all observations up to 24 months (i.e., without missing observations) and the results were similar to those from all observations (data not shown). Thus, the missing data should be “at random” [9] Also, the birth measures obtained from medical records may not be ideal despite of the high number of the participating hospitals (which were all provincial or large tertiary maternity and child hospitals). Thirdly, this was a convenience sample without specific entry criteria as in the WHO study.

## Conclusions

The growth curves in this study represent the growth pattern of today’s normal Chinese children, and may provide references for evaluation of the individual growth status of children growing up in modern China.

## Additional file

Additional file 1 **Table S1.** The overall information of 6 prospective birth cohorts in China for description of growth pattern in this study. **Table S2.** the numbers of longitudinal anthropometric measurements used for generating growth curves from 2174 infant boys and 2077 infant girls. **Table S3.** the numbers of longitudinal measures used for generating weight-for-length curves from 2174 infant boys and 2077 infant girls. **Table S4.** The 3rd, 10th, 50th, 90th and 97th percentiles of growth measures by age among 2174 healthy infant boys. **Table S5.** The 3rd, 10th, 50th, 90th and 97th percentiles of growth measures by age among 2077 healthy infant girls. **Table S6.** The difference in the 3rd, 10th, 50th, 90th and 97th percentiles for growth measures between the raw data of 2174 healthy infant boys and the corresponding WHO standards. **Table S7.** The difference between the 3rd, 10th, 50th, 90th and 97th percentiles for growth measures of the raw data in 2077 healthy infant girls and the corresponding WHO standards by age. **Table S8.** Distribution of weight by length among 2174 infant boys and 2077 infant girls. **Table S9.** The difference between the percentile values of the raw data of among 2174 infant boys and 2077 infant girls and the corresponding WHO standards. (DOC 485 kb)

## Abbreviations

ART: Assisted reproductive technologies
CBCC: China Birth Cohort Consortium
GDM: Gestational diabetes mellitus
IADPSG: International Association of Diabetes and Pregnancy Study Groups
MGRS: Multicenter Growth Reference Study
WHO: World Health Organization

## References

[1] Blake-Lamb TL, Locks LM, Perkins ME, Woo Baidal JA, Cheng ER, Taveras EM (2016). Interventions for childhood obesity in the first 1,000 days a systematic review. Am J Prev Med.

[2] de Onis M, Onyango A, Borghi E, Siyam A, Blossner M, Lutter C, Group WHOMGRS (2012). Worldwide implementation of the WHO child growth standards. Public Health Nutr.

[3] Zong XN, Li H (2013). Construction of a new growth references for China based on urban Chinese children: comparison with the WHO growth standards. PLoS One.

[4] Cole TJ (2003). The secular trend in human physical growth: a biological view. Econ Hum Biol.

[5] Huang X, Chang J, Feng W, Xu Y, Xu T, Tang H, Wang H, Pan X (2016). Development of a new growth standard for breastfed Chinese infants: what is the difference from the WHO growth standards?. PLoS One.

[6] Tanner JM, Whitehouse RH, Takaishi M (1966). Standards from birth to maturity for height, weight, height velocity, and weight velocity: British children, 1965. II. Arch Dis Child.

[7] Xu F, Qiu L, Binns CW, Liu X (2009). Breastfeeding in China: a review. Int Breastfeed J.

[8] American Diabetes A (2013). Diagnosis and classification of diabetes mellitus. Diabetes Care.

[9] Croy CD, Novins DK (2005). Methods for addressing missing data in psychiatric and developmental research. J Am Acad Child Adolesc Psychiatry.

[10] Gillman MW, Rifas-Shiman S, Berkey CS, Field AE, Colditz GA (2003). Maternal gestational diabetes, birth weight, and adolescent obesity. Pediatrics.

[11] Paauw ND, van Rijn BB, Lely AT, Joles JA. Pregnancy as a critical window for blood pressure regulation in mother and child: programming and reprogramming. Acta Physiol (Oxf). 2016. 10.1111/apha.12702 27124608

[12] Ogland B, Vatten LJ, Romundstad PR, Nilsen ST, Forman MR (2009). Pubertal anthropometry in sons and daughters of women with preeclamptic or normotensive pregnancies. Arch Dis Child.

[13] Cole TJ, Green PJ (1992). Smoothing reference centile curves: the LMS method and penalized likelihood. Stat Med.

[14] Borghi E, de Onis M, Garza C, Van den Broeck J, Frongillo EA, Grummer-Strawn L, Van Buuren S, Pan H, Molinari L, Martorell R (2006). Construction of the World Health Organization child growth standards: selection of methods for attained growth curves. Stat Med.

[15] Garza C, Borghi E, Onyango AW, de Onis M, Group WHOMGRS (2013). Parental height and child growth from birth to 2 years in the WHO multicentre growth reference study. Matern Child Nutr.

[16] Aman B, Pulungan MJ, Batubara JRL, Hermanussen M (2018). Indonesian national synthetic growth charts. Acta Scientific Paediatrics.

[17] Dang S, Yan H, Wang D (2014). Implication of World Health Organization growth standards on estimation of malnutrition in young Chinese children: two examples from rural western China and the Tibet region. J Child Health Care.

[18] Hui LL, Schooling CM, Cowling BJ, Leung SS, Lam TH, Leung GM (2008). Are universal standards for optimal infant growth appropriate? Evidence from a Hong Kong Chinese birth cohort. Arch Dis Child.

[19] Natale V, Rajagopalan A (2014). Worldwide variation in human growth and the World Health Organization growth standards: a systematic review. BMJ Open.

[20] Tanner JM, Whitehouse RH, Takaishi M (1966). Standards from birth to maturity for height, weight, height velocity, and weight velocity: British children, 1965. I. Arch Dis Child.

[21] Acuna-Hidalgo R, Veltman JA, Hoischen A (2016). New insights into the generation and role of de novo mutations in health and disease. Genome Biol.

[22] Tanner JM, Whitehouse RH (1976). Clinical longitudinal standards for height, weight, height velocity, weight velocity, and stages of puberty. Arch Dis Child.

[23] Wachholder A, Hauspie RC (1986). Clinical standards for growth in height of Belgian boys and girls, aged 2 to 18. International Journal of Anthropology.

