# Supplementary material for: Growth patterns from birth to 24 months in Chinese children: a birth cohorts study across China
Source: BMC Pediatr. 2018 Nov 5;18:344. doi: 10.1186/s12887-018-1328-z (PMC6217782; doi:10.1186/s12887-018-1328-z)
Supplement: Supplementary file 1 — Table S1. The overall information of 6 prospective birth cohorts in China for description of growth pattern in this study. Table S2. the numbers of longitudinal anthropometric measurements used for generating growth curves from 2174 infant boys and 2077 infant girls. Table S3. the numbers of longitudinal measures used for generating weight-for-length curves from 2174 infant boys and 2077 infant girls. Table S4. The 3rd, 10th, 50th, 90th and 97th percentiles of growth measures by age among 2174 healthy infant boys. Table S5. The 3rd, 10th, 50th, 90th and 97th percentiles of growth measures by age among 2077 healthy infant girls. Table S6. The difference in the 3rd, 10th, 50th, 90th and 97th percentiles for growth measures between the raw data of 2174 healthy infant boys and the corresponding WHO standards. Table S7. The difference between the 3rd, 10th, 50th, 90th and 97th percentiles for growth measures of the raw data in 2077 healthy infant girls and the corresponding WHO standards by age. Table S8. Distribution of weight by length among 2174 infant boys and 2077 infant girls. Table S9. The difference between the percentile values of the raw data of among 2174 infant boys and 2077 infant girls and the corresponding WHO standards. (DOC 485 kb) [file 12887_2018_1328_MOESM1_ESM.doc]

**Additional file 1:**

| Table S1_1. The overall information of 6 prospective birth cohorts in China for description of growth pattern in this study. | | | | | | | | | |
| --- | --- | --- | --- | --- | --- | --- | --- | --- | --- |
| **Cohort**  **Name** | **Location** | **Starting**  **Date** | **No. of subjects**  **recruited** | **Entry time** | **Target No.**  **Of subjects** | **Ending**  **recruitment**  **Date** | **Child follow-up**  **schedule** | **Contributed Sample size in this report** | **Child follow-up**  **Completed**  **or ongoing** |
| Shanghai Birth Cohort | Shanghai | 4-2013 | 5000 couples | preconception  or ≤ 16 weeks | 5000 couples | 4-2016 | 6 weeks, 6, 12, and 24 months, 3, 5, 6, 10 yr | 830 | 6 weeks , 3,6,9, 12, 18, and 24 months |
| SCMC Birth Cohort | Shanghai | 8-2012 | 277couples | during pregnancy | 300 couples | 7-2013 | 6 weeks, 3, 6, 9 mo, 1, 2, 3, 4, 5, 6, 7, 8, 9, 10 , 11, 12, 13, 14, 15, 16, 17, 18yr | 261 | 6 weeks, 3, 6, 9, 12, 18, 24 months |
| Anhui Birth Cohort | Ma’anshan, Anhui | 5-2013 | 3474 pregnant women within 14 weeks | ≤ 16 weeks | 3000 women | 9-2014 | 3m, 6m, 9m, 12m, and 18 m; 2, 3, 4, 5, 6y | 1062 | 6 weeks; 3, 6, 9, 12, 18 and 24 months |
| Healthy Baby Cohort | Wuhan | 9-2012 | 20,000 couples | during pregnancy | 20,000 couples | 10-2014 | 1, 3, 6 mo, 1, 2,  3, 6, 12 yr | 1000 | 1, 3, 6, 8, 12, 18, 24 months |
| NJMU Growth and Development Cohort | Jiangsu | 6-2014 | 2000 couples | preconception or early pregnancy | 4000 couples | 12-2017 | 6 weeks, 3m, 6m, 1, 2, 3yr | 1000 | 6 weeks, 3m, 6m, 8, 12 months |
| Born in Guangzhou Cohort Study | Guangzhou | 02-2012 | 21,000 Mother-child pairs | < 20 weeks | 30,000 Mother-child pairs | 12-2018 | 6 weeks, 6 m, 1, 3, 6, 10, 14, 16, 18yr | 999 | 6 weeks, 6 and 12 months |
| SCMC: Shanghai Children's Medical Center; NJMU: Nan-Jing Medical University. | | | | | | | | | |

| Table S1_2. The study objectives of 6 prospective birth cohorts in China which participated in this study. | |
| --- | --- |
| **Cohort**  **Name** | Study Objectives |
| Shanghai Birth Cohort | To examine the effects of genetic, environmental and behavioral factors on reproductive health, pregnancy outcomes, child growth and development, risks of diseases and mechanism of action. |
| SCMC Birth Cohort | To examine the effects of social and behavioral factors on child growth and development. |
| Anhui Birth Cohort | To examine the effects of environmental exposure, endocrine and metabolic disorders during pregnancy on birth outcomes and child development. |
| Healthy Baby Cohort | To examine the effects of adverse environmental exposure on maternal and child health.  To provide evidence for disease prevention and interventions through the exploration of associations between child growth and diseases as well as biological mechanisms. |
| NJMU Growth and Development Cohort | To examine the effects of environmental factors in early life, genetics, epigenetics, nutrition and behaviors on reproductive health, pregnancy outcomes, child growth and development, and risks of diseases. |
| Born in Guangzhou Cohort Study | To examine main factors affecting maternal and child health in Guangzhou area, including social, economic, cultural, biological and environmental factors and their biological mechanisms.  To provide evidence for interventions, health services and policies to improve maternal and child health. |
| SCMC: Shanghai Children's Medical Center; NJMU: Nan-Jing Medical University. | |

| Table S2. the numbers of longitudinal anthropometric measurements used for generating growth curves from 2174 infant boys and 2077 infant girls. | | | | | | | | | | | |
| --- | --- | --- | --- | --- | --- | --- | --- | --- | --- | --- | --- |
| Age (Months) | Weight | |  | Length | |  | BMI | |  | Head Circumference | |
|  | Boys | Girls |  | Boys | Girls |  | Boys | Girls |  | Boys | Girls |
| 0* | 2321 | 2216 |  | 2321 | 2216 |  | 2321 | 2216 |  | 1109 | 1063 |
| 1- | 1829 | 1746 |  | 1829 | 1746 |  | 1829 | 1746 |  | 1789 | 1710 |
| 2- | 358 | 325 |  | 358 | 325 |  | 358 | 325 |  | 311 | 292 |
| 3- | 1284 | 1204 |  | 1284 | 1204 |  | 1284 | 1204 |  | 1261 | 1187 |
| 4- | 239 | 241 |  | 239 | 241 |  | 239 | 241 |  | 185 | 197 |
| 5- | 302 | 303 |  | 302 | 303 |  | 302 | 303 |  | 296 | 299 |
| 6- | 1777 | 1674 |  | 1777 | 1674 |  | 1777 | 1674 |  | 1704 | 1610 |
| 7- | 145 | 160 |  | 145 | 160 |  | 145 | 160 |  | 140 | 155 |
| 8- | 780 | 730 |  | 780 | 730 |  | 780 | 730 |  | 748 | 701 |
| 9- | 758 | 672 |  | 758 | 672 |  | 758 | 672 |  | 702 | 624 |
| 10- | 30 | 63 |  | 30 | 63 |  | 30 | 63 |  | 25 | 54 |
| 11- | 236 | 231 |  | 236 | 231 |  | 236 | 231 |  | 231 | 224 |
| 12- | 1606 | 1538 |  | 1606 | 1538 |  | 1606 | 1538 |  | 1517 | 1462 |
| 13- | 216 | 213 |  | 216 | 213 |  | 216 | 213 |  | 209 | 211 |
| 14- | 41 | 47 |  | 41 | 47 |  | 41 | 47 |  | 40 | 47 |
| 15- | 33 | 42 |  | 33 | 42 |  | 33 | 42 |  | 28 | 38 |
| 16- | 12 | 13 |  | 12 | 13 |  | 12 | 13 |  | 12 | 13 |
| 17- | 28 | 28 |  | 28 | 28 |  | 28 | 28 |  | 27 | 25 |
| 18- | 1096 | 1004 |  | 1096 | 1004 |  | 1096 | 1004 |  | 1011 | 932 |
| 19- | 109 | 91 |  | 109 | 91 |  | 109 | 91 |  | 106 | 90 |
| 20- | 12 | 18 |  | 12 | 18 |  | 12 | 18 |  | 11 | 18 |
| 21- | 10 | 13 |  | 10 | 13 |  | 10 | 13 |  | 9 | 11 |
| 22- | 4 | 2 |  | 4 | 2 |  | 4 | 2 |  | 2 | 2 |
| 23- | 124 | 103 |  | 124 | 103 |  | 124 | 103 |  | 124 | 102 |
| 24- | 988 | 926 |  | 988 | 926 |  | 988 | 926 |  | 963 | 905 |
| 25- | 155 | 145 |  | 155 | 145 |  | 155 | 145 |  | 155 | 145 |
| 26- | 23 | 27 |  | 23 | 27 |  | 23 | 27 |  | 23 | 26 |
| 27 to <28 | 3 | 4 |  | 3 | 4 |  | 3 | 4 |  | 3 | 4 |

* In the first month, some infants were measured twice due to that they came for the follow-up visits ahead of schedule.

| Table S3. the numbers of longitudinal measures used for generating weight-for-length curves from 2174 infant boys and 2077 infant girls. | | | | | | | | | | |
| --- | --- | --- | --- | --- | --- | --- | --- | --- | --- | --- |
| Length (Cm) | Boys | Girls |  | Length (Cm) | Boys | Girls |  | Length (Cm) | Boys | Girls |
| 45 | 6 | 5 |  | 65 | 276 | 303 |  | 85 | 215 | 201 |
| 46 | 13 | 29 |  | 66 | 325 | 357 |  | 86 | 233 | 197 |
| 47 | 40 | 73 |  | 67 | 344 | 436 |  | 87 | 222 | 200 |
| 48 | 161 | 250 |  | 68 | 422 | 457 |  | 88 | 243 | 192 |
| 49 | 272 | 319 |  | 69 | 463 | 409 |  | 89 | 176 | 166 |
| 50 | 1026 | 985 |  | 70 | 477 | 463 |  | 90 | 183 | 129 |
| 51 | 374 | 315 |  | 71 | 464 | 380 |  | 91 | 134 | 105 |
| 52 | 255 | 200 |  | 72 | 479 | 420 |  | 92 | 132 | 66 |
| 53 | 184 | 246 |  | 73 | 466 | 437 |  | 93 | 86 | 38 |
| 54 | 258 | 349 |  | 74 | 416 | 439 |  | 94 | 52 | 14 |
| 55 | 393 | 435 |  | 75 | 422 | 393 |  | 95 | 27 | 8 |
| 56 | 375 | 352 |  | 76 | 456 | 388 |  | 96 | 11 | 5 |
| 57 | 352 | 266 |  | 77 | 416 | 306 |  | 97 | 4 | 5 |
| 58 | 293 | 221 |  | 78 | 327 | 251 |  | 98 | 9 | 2 |
| 59 | 222 | 197 |  | 79 | 267 | 183 |  | 99 | 0 | 2 |
| 60 | 243 | 294 |  | 80 | 221 | 215 |  | 100 | 0 | 1 |
| 61 | 244 | 305 |  | 81 | 205 | 212 |  | 101 | 1 | 0 |
| 62 | 337 | 342 |  | 82 | 250 | 216 |  |  |  |  |
| 63 | 309 | 318 |  | 83 | 214 | 196 |  |  |  |  |
| 64 | 326 | 284 |  | 84 | 196 | 202 |  |  |  |  |

| Table S4. The 3^rd^, 10^th^, 50^th^, 90^th^ and 97^th^ percentiles of growth measures by age among 2174 healthy infant boys. | | | | | | | | | | | | | | | | | | | | | | | |
| --- | --- | --- | --- | --- | --- | --- | --- | --- | --- | --- | --- | --- | --- | --- | --- | --- | --- | --- | --- | --- | --- | --- | --- |
| Age (months) | Boy | | | | | | | | | | | | | | | | | | | | | | |
|  | Weight (kg) | | | | |  | Length (cm) | | | | |  | head circumference (cm) | | | | |  | BMI (kg/m^2^) | | | | |
|  | P3 | P10 | P50 | P90 | P97 |  | P3 | P10 | P50 | P90 | P97 |  | P3 | P10 | P50 | P90 | P97 |  | P3 | P10 | P50 | P90 | P97 |
| 0 | 2.70 | 2.90 | 3.42 | 4.11 | 5.00 |  | 48.0 | 49.0 | 50.0 | 53.0 | 56.0 |  | 32.1 | 33.0 | 34.0 | 37.1 | 38.6 |  | 11.2 | 12.0 | 13.5 | 15.4 | 16.5 |
| 1- | 4.00 | 4.30 | 5.00 | 5.80 | 6.27 |  | 52.5 | 54.0 | 56.1 | 59.0 | 60.0 |  | 36.0 | 36.5 | 38.0 | 39.5 | 40.0 |  | 13.4 | 14.1 | 15.8 | 17.5 | 18.8 |
| 2- | 5.30 | 5.60 | 6.50 | 7.70 | 8.25 |  | 57.0 | 58.0 | 61.5 | 64.8 | 66.0 |  | 38.0 | 38.7 | 40.4 | 42.1 | 42.8 |  | 14.7 | 15.6 | 17.4 | 19.6 | 20.8 |
| 3- | 5.70 | 6.10 | 7.00 | 8.10 | 8.80 |  | 59.0 | 60.0 | 63.0 | 65.2 | 66.5 |  | 38.5 | 39.4 | 40.9 | 42.2 | 43.0 |  | 15.0 | 15.9 | 17.8 | 20.0 | 21.4 |
| 4- | 6.40 | 6.90 | 7.80 | 8.75 | 9.60 |  | 60.8 | 62.6 | 65.6 | 68.0 | 69.2 |  | 39.0 | 40.0 | 42.0 | 44.0 | 44.5 |  | 15.8 | 16.6 | 18.3 | 20.2 | 20.8 |
| 5- | 6.60 | 7.20 | 8.33 | 9.55 | 10.50 |  | 63.7 | 65.0 | 68.0 | 71.2 | 72.5 |  | 40.8 | 41.5 | 43.2 | 45.0 | 46.0 |  | 15.0 | 16.0 | 18.1 | 20.1 | 21.2 |
| 6- | 7.00 | 7.50 | 8.60 | 10.00 | 10.70 |  | 65.0 | 66.0 | 69.0 | 72.0 | 73.1 |  | 41.2 | 42.0 | 43.5 | 45.0 | 46.0 |  | 15.3 | 16.2 | 18.1 | 20.3 | 21.4 |
| 7- | 7.65 | 8.00 | 9.20 | 10.50 | 11.50 |  | 66.5 | 68.0 | 71.2 | 75.0 | 77.0 |  | 42.5 | 43.0 | 44.3 | 46.6 | 47.2 |  | 15.7 | 16.6 | 18.1 | 20.0 | 21.4 |
| 8- | 7.90 | 8.40 | 9.50 | 10.83 | 11.65 |  | 68.7 | 69.9 | 72.0 | 75.0 | 77.0 |  | 42.8 | 43.5 | 45.0 | 46.7 | 47.8 |  | 15.6 | 16.4 | 18.2 | 20.3 | 21.3 |
| 9 to <10 | 8.00 | 8.50 | 9.70 | 11.20 | 12.20 |  | 69.0 | 71.0 | 73.5 | 76.6 | 78.0 |  | 43.3 | 43.8 | 45.2 | 47.0 | 47.7 |  | 15.3 | 16.2 | 17.9 | 19.9 | 21.3 |
| 11- | 8.00 | 8.70 | 10.00 | 11.40 | 12.30 |  | 71.0 | 72.6 | 76.0 | 79.1 | 81.0 |  | 44.0 | 44.5 | 46.0 | 47.6 | 48.6 |  | 15.0 | 15.6 | 17.2 | 19.1 | 20.1 |
| 12- | 8.40 | 9.05 | 10.30 | 11.90 | 12.85 |  | 72.2 | 74.0 | 77.0 | 80.0 | 82.0 |  | 44.0 | 44.9 | 46.2 | 48.0 | 48.6 |  | 15.0 | 15.8 | 17.5 | 19.4 | 20.4 |
| 13 to <14 | 8.80 | 9.40 | 10.70 | 12.30 | 13.10 |  | 72.7 | 74.8 | 78.0 | 81.2 | 82.4 |  | 44.5 | 45.0 | 47.0 | 48.3 | 49.1 |  | 15.2 | 15.9 | 17.6 | 19.6 | 21.2 |
| 18- | 9.80 | 10.30 | 11.70 | 13.20 | 14.20 |  | 79.0 | 80.5 | 84.0 | 87.0 | 89.0 |  | 45.3 | 46.0 | 47.5 | 49.0 | 50.0 |  | 14.5 | 15.2 | 16.8 | 18.4 | 19.2 |
| 19 to <20 | 10.00 | 10.30 | 12.00 | 13.70 | 14.30 |  | 80.0 | 81.0 | 84.0 | 87.5 | 89.0 |  | 45.7 | 46.0 | 47.5 | 49.5 | 50.0 |  | 14.5 | 15.2 | 17.1 | 18.6 | 19.0 |
| 23- | 10.90 | 11.30 | 12.90 | 14.90 | 16.30 |  | 84.1 | 86.0 | 89.0 | 93.0 | 94.3 |  | 46.2 | 47.1 | 48.8 | 50.6 | 51.6 |  | 14.1 | 14.9 | 16.4 | 17.9 | 18.9 |
| 24- | 10.80 | 11.40 | 13.00 | 14.70 | 15.60 |  | 84.0 | 86.0 | 89.5 | 93.0 | 95.0 |  | 46.4 | 47.0 | 48.5 | 50.2 | 51.0 |  | 14.0 | 14.7 | 16.1 | 17.7 | 18.5 |
| 25 to <26 | 10.80 | 11.70 | 13.10 | 15.40 | 16.50 |  | 84.0 | 86.0 | 90.0 | 93.1 | 95.0 |  | 46.1 | 47.0 | 48.7 | 50.8 | 51.8 |  | 14.2 | 15.0 | 16.5 | 17.9 | 18.7 |
| * data are presented if sample size > 100 observations. | | | | | | | | | | | | | | | | | | | | | | | |

| Table S5. The 3^rd^, 10^th^, 50^th^, 90^th^ and 97^th^ percentiles of growth measures by age among 2077 healthy infant girls. | | | | | | | | | | | | | | | | | | | | | | | |
| --- | --- | --- | --- | --- | --- | --- | --- | --- | --- | --- | --- | --- | --- | --- | --- | --- | --- | --- | --- | --- | --- | --- | --- |
| Age (months) | Girl | | | | | | | | | | | | | | | | | | | | | | |
|  | Weight (kg) | | | | |  | Length (cm) | | | | |  | head circumference (cm) | | | | |  | BMI (kg/m^2^) | | | | |
|  | P3 | P10 | P50 | P90 | P97 |  | P3 | P10 | P50 | P90 | P97 |  | P3 | P10 | P50 | P90 | P97 |  | P3 | P10 | P50 | P90 | P97 |
| 0 | 2.62 | 2.85 | 3.33 | 4.04 | 4.90 |  | 47.0 | 48.0 | 50.0 | 52.0 | 55.8 |  | 32.1 | 33.0 | 34.0 | 37.0 | 38.7 |  | 11.2 | 11.9 | 13.4 | 15.3 | 16.4 |
| 1- | 3.80 | 4.10 | 4.70 | 5.50 | 5.90 |  | 51.9 | 53.0 | 55.1 | 58.0 | 59.0 |  | 35.5 | 36.0 | 37.4 | 39.0 | 39.4 |  | 13.2 | 13.9 | 15.4 | 17.2 | 18.2 |
| 2- | 4.70 | 5.20 | 6.20 | 7.30 | 8.00 |  | 55.3 | 57.0 | 60.0 | 63.5 | 65.2 |  | 37.0 | 38.0 | 39.8 | 41.5 | 42.5 |  | 14.4 | 15.4 | 16.9 | 19.2 | 20.3 |
| 3- | 5.40 | 5.80 | 6.60 | 7.60 | 8.15 |  | 58.0 | 59.0 | 62.0 | 64.0 | 65.5 |  | 38.0 | 38.7 | 40.0 | 41.5 | 42.3 |  | 14.8 | 15.5 | 17.4 | 19.5 | 20.5 |
| 4- | 6.00 | 6.30 | 7.20 | 8.25 | 8.70 |  | 60.0 | 61.0 | 63.8 | 66.3 | 68.0 |  | 38.5 | 39.2 | 41.0 | 42.7 | 44.0 |  | 15.1 | 16.0 | 17.9 | 20.0 | 20.9 |
| 5- | 6.30 | 6.60 | 7.90 | 9.00 | 9.70 |  | 62.2 | 64.0 | 67.0 | 70.0 | 71.6 |  | 40.0 | 41.0 | 42.5 | 44.3 | 45.5 |  | 14.7 | 15.6 | 17.6 | 19.6 | 20.9 |
| 6- | 6.60 | 7.00 | 8.10 | 9.35 | 10.20 |  | 63.5 | 65.0 | 67.7 | 70.5 | 72.0 |  | 40.2 | 41.1 | 42.8 | 44.2 | 45.0 |  | 15.0 | 15.8 | 17.7 | 20.0 | 21.1 |
| 7- | 6.70 | 7.05 | 8.50 | 10.00 | 10.60 |  | 64.0 | 66.1 | 70.0 | 73.4 | 74.5 |  | 40.6 | 42.0 | 43.8 | 46.0 | 47.2 |  | 14.7 | 15.4 | 17.5 | 19.4 | 20.9 |
| 8- | 7.50 | 7.93 | 9.00 | 10.40 | 11.20 |  | 67.0 | 68.0 | 71.0 | 74.0 | 76.0 |  | 42.0 | 42.8 | 44.0 | 45.8 | 46.9 |  | 15.2 | 16.1 | 17.9 | 20.0 | 21.1 |
| 9 to < 10 | 7.60 | 8.00 | 9.10 | 10.60 | 11.65 |  | 68.0 | 69.3 | 72.3 | 75.0 | 77.0 |  | 42.3 | 43.0 | 44.3 | 46.0 | 47.0 |  | 15.1 | 15.9 | 17.5 | 19.6 | 20.8 |
| 11- | 7.20 | 8.00 | 9.25 | 11.00 | 11.70 |  | 70.0 | 71.0 | 74.3 | 78.0 | 80.0 |  | 42.5 | 43.5 | 45.0 | 47.0 | 48.2 |  | 14.1 | 15.0 | 16.8 | 18.7 | 19.8 |
| 12- | 8.00 | 8.50 | 9.85 | 11.30 | 12.20 |  | 71.0 | 72.8 | 76.0 | 79.0 | 80.7 |  | 43.0 | 44.0 | 45.5 | 47.0 | 48.0 |  | 14.7 | 15.5 | 17.2 | 19.0 | 20.1 |
| 13 to <14 | 8.30 | 9.00 | 10.00 | 11.60 | 12.60 |  | 72.1 | 73.8 | 76.5 | 80.2 | 82.0 |  | 43.3 | 44.0 | 46.0 | 47.3 | 48.1 |  | 14.9 | 15.5 | 17.0 | 19.0 | 20.3 |
| 18- | 9.30 | 9.80 | 11.20 | 12.60 | 13.40 |  | 77.8 | 79.0 | 82.2 | 86.0 | 87.8 |  | 44.5 | 45.4 | 47.0 | 48.2 | 49.0 |  | 14.2 | 14.9 | 16.4 | 18.0 | 18.8 |
| 19 to <20** | 9.20 | 10.00 | 11.25 | 12.90 | 14.00 |  | 77.3 | 80.0 | 83.0 | 86.8 | 88.0 |  | 44.8 | 45.0 | 47.0 | 48.6 | 50.0 |  | 14.2 | 15.0 | 16.3 | 18.1 | 19.6 |
| 23- | 10.00 | 10.25 | 12.40 | 14.00 | 14.70 |  | 82.9 | 84.4 | 87.3 | 91.9 | 92.9 |  | 45.8 | 46.5 | 48.0 | 49.4 | 50.0 |  | 13.9 | 14.3 | 16.0 | 17.6 | 18.1 |
| 24- | 10.23 | 10.90 | 12.40 | 14.00 | 15.20 |  | 83.0 | 84.8 | 88.0 | 92.0 | 93.8 |  | 45.6 | 46.5 | 48.0 | 49.5 | 50.0 |  | 13.8 | 14.5 | 15.9 | 17.6 | 18.7 |
| 25 to <26 | 10.60 | 11.20 | 12.60 | 14.30 | 15.40 |  | 84.5 | 85.2 | 89.0 | 92.2 | 95.3 |  | 46.0 | 46.4 | 48.0 | 49.7 | 50.5 |  | 13.6 | 14.5 | 16.1 | 17.7 | 18.5 |
| * data are presented if sample size > 100 observations. ** the sample size in this age group was 90 or 91. | | | | | | | | | | | | | | | | | | | | | | | |

| Table S6. The difference in the 3^rd^, 10^th^, 50^th^, 90^th^ and 97^th^ percentiles for growth measures between the raw data of 2174 healthy infant boys and the corresponding WHO standards. | | | | | | | | | | | | | | | | | | | | | | | |
| --- | --- | --- | --- | --- | --- | --- | --- | --- | --- | --- | --- | --- | --- | --- | --- | --- | --- | --- | --- | --- | --- | --- | --- |
| Age (months) | Boy difference in the percentiles for growth measures (The present study – WHO) | | | | | | | | | | | | | | | | | | | | | | |
|  | Weight (kg) | | | | |  | Length (cm) | | | | |  | head circumference (cm) | | | | |  | BMI (kg/m^2^) | | | | |
|  | P3 | P10 | P50 | P90 | P97 |  | P3 | P10 | P50 | P90 | P97 |  | P3 | P10 | P50 | P90 | P97 |  | P3 | P10 | P50 | P90 | P97 |
| 0 | 0.2 | 0.1 | 0.1 | 0.1 | 0.7 |  | 1.7 | 1.5 | 0.1 | 0.7 | 2.6 |  | 0.0 | 0.2 | -0.5 | 1.0 | 1.7 |  | -0.1 | 0.1 | 0.1 | 0.2 | 0.4 |
| 1- | 0.6 | 0.5 | 0.5 | 0.5 | 0.6 |  | 1.4 | 1.8 | 1.4 | 1.8 | 1.6 |  | 0.9 | 0.7 | 0.7 | 0.7 | 0.5 |  | 0.8 | 0.8 | 0.9 | 0.8 | 1.2 |
| 2- | 0.9 | 0.9 | 0.9 | 1.2 | 1.3 |  | 2.3 | 2.1 | 3.1 | 3.8 | 3.8 |  | 1.1 | 1.1 | 1.3 | 1.5 | 1.5 |  | 0.9 | 1.0 | 1.1 | 1.4 | 1.6 |
| 3- | 0.6 | 0.6 | 0.6 | 0.7 | 0.9 |  | 1.4 | 1.2 | 1.6 | 1.2 | 1.2 |  | 0.2 | 0.4 | 0.4 | 0.2 | 0.3 |  | 0.6 | 0.7 | 0.9 | 1.2 | 1.6 |
| 4- | 0.8 | 0.9 | 0.8 | 0.7 | 1.0 |  | 0.8 | 1.4 | 1.7 | 1.4 | 1.4 |  | -0.4 | -0.1 | 0.4 | 0.8 | 0.6 |  | 1.1 | 1.2 | 1.1 | 1.1 | 0.7 |
| 5- | 0.5 | 0.7 | 0.8 | 1.0 | 1.3 |  | 1.8 | 1.8 | 2.1 | 2.6 | 2.6 |  | 0.5 | 0.5 | 0.6 | 0.9 | 1.2 |  | 0.2 | 0.4 | 0.8 | 0.9 | 1.0 |
| 6- | 0.6 | 0.6 | 0.7 | 0.9 | 1.0 |  | 1.4 | 1.1 | 1.4 | 1.6 | 1.5 |  | 0.2 | 0.2 | 0.2 | 0.1 | 0.4 |  | 0.4 | 0.6 | 0.8 | 1.0 | 1.1 |
| 7- | 1.0 | 0.8 | 0.9 | 1.0 | 1.3 |  | 1.4 | 1.6 | 2.0 | 3.1 | 3.8 |  | 0.8 | 0.6 | 0.3 | 1.0 | 0.9 |  | 0.8 | 1.0 | 0.8 | 0.7 | 1.1 |
| 8- | 0.9 | 0.9 | 0.9 | 0.9 | 1.2 |  | 2.2 | 2.1 | 1.4 | 1.6 | 2.3 |  | 0.6 | 0.6 | 0.5 | 0.6 | 0.9 |  | 0.7 | 0.8 | 0.9 | 1.1 | 1.1 |
| 9 to <10 | 0.8 | 0.8 | 0.8 | 1.0 | 1.3 |  | 1.3 | 1.9 | 1.5 | 1.8 | 1.8 |  | 0.7 | 0.4 | 0.2 | 0.4 | 0.3 |  | 0.5 | 0.7 | 0.7 | 0.8 | 1.2 |
| 11- | 0.3 | 0.5 | 0.6 | 0.6 | 0.8 |  | 0.8 | 1.0 | 1.5 | 1.6 | 2.1 |  | 0.6 | 0.4 | 0.2 | 0.2 | 0.4 |  | 0.4 | 0.3 | 0.3 | 0.3 | 0.3 |
| 12- | 0.6 | 0.7 | 0.7 | 0.8 | 1.0 |  | 0.9 | 1.3 | 1.3 | 1.2 | 1.8 |  | 0.4 | 0.5 | 0.1 | 0.3 | 0.1 |  | 0.5 | 0.6 | 0.7 | 0.7 | 0.8 |
| 13 to <14 | 0.8 | 0.8 | 0.8 | 0.9 | 1.0 |  | 0.3 | 1.0 | 1.1 | 1.2 | 0.9 |  | 0.6 | 0.3 | 0.7 | 0.3 | 0.3 |  | 0.8 | 0.8 | 0.9 | 1.1 | 1.7 |
| 18- | 0.9 | 0.8 | 0.8 | 0.6 | 0.7 |  | 1.8 | 1.7 | 1.7 | 1.3 | 1.7 |  | 0.4 | 0.3 | 0.1 | -0.1 | 0.1 |  | 0.5 | 0.6 | 0.7 | 0.5 | 0.4 |
| 19 to <20 | 1.0 | 0.6 | 0.9 | 0.8 | 0.6 |  | 1.9 | 1.3 | 0.8 | 0.7 | 0.6 |  | 0.7 | 0.2 | 0.0 | 0.3 | 0.0 |  | 0.6 | 0.6 | 1.0 | 0.8 | 0.3 |
| 23- | 1.2 | 1.0 | 0.9 | 1.1 | 1.5 |  | 2.8 | 2.9 | 2.1 | 2.2 | 1.7 |  | 0.6 | 0.7 | 0.7 | 0.7 | 0.9 |  | 0.4 | 0.6 | 0.6 | 0.4 | 0.5 |
| 24- | 1.0 | 0.9 | 0.8 | 0.6 | 0.5 |  | 1.9 | 2.1 | 1.7 | 1.3 | 1.4 |  | 0.7 | 0.5 | 0.2 | 0.2 | 0.2 |  | 0.3 | 0.4 | 0.4 | 0.3 | 0.2 |
| 25 to <26 | 0.8 | 1.0 | 0.7 | 1.1 | 1.2 |  | 1.9 | 2.0 | 2.0 | 1.1 | 1.2 |  | 0.3 | 0.4 | 0.3 | 0.7 | 0.9 |  | 0.3 | 0.5 | 0.5 | 0.2 | 0.1 |
| * data are presented if sample size > 100 observations. The difference is the percentile values in this study minus the WHO standards | | | | | | | | | | | | | | | | | | | | | | | |

| Table S7. The difference between the 3^rd^, 10^th^, 50^th^, 90^th^ and 97^th^ percentiles for growth measures of the raw data in 2077 healthy infant girls and the corresponding WHO standards by age. | | | | | | | | | | | | | | | | | | | | | | | |
| --- | --- | --- | --- | --- | --- | --- | --- | --- | --- | --- | --- | --- | --- | --- | --- | --- | --- | --- | --- | --- | --- | --- | --- |
| Age (months) | Girl difference in the percentiles for growth measures (The present study – WHO) | | | | | | | | | | | | | | | | | | | | | | |
|  | Weight (kg) | | | | |  | Length (cm) | | | | |  | head circumference (cm) | | | | |  | BMI (kg/m^2^) | | | | |
|  | P3 | P10 | P50 | P90 | P97 |  | P3 | P10 | P50 | P90 | P97 |  | P3 | P10 | P50 | P90 | P97 |  | P3 | P10 | P50 | P90 | P97 |
| 0 | 0.2 | 0.2 | 0.1 | 0.1 | 0.7 |  | 1.4 | 1.2 | 0.9 | 0.5 | 3.1 |  | 0.4 | 0.6 | 0.1 | 1.6 | 2.6 |  | 0.0 | 0.1 | 0.1 | 0.3 | 0.5 |
| 1- | 0.6 | 0.6 | 0.5 | 0.5 | 0.5 |  | 1.9 | 1.8 | 1.4 | 1.8 | 1.6 |  | 1.2 | 1.0 | 0.9 | 1.0 | 0.6 |  | 1.1 | 1.0 | 0.8 | 0.8 | 0.9 |
| 2- | 0.7 | 0.9 | 1.1 | 1.3 | 1.5 |  | 2.1 | 2.5 | 2.9 | 3.8 | 4.3 |  | 1.0 | 1.3 | 1.5 | 1.7 | 2.0 |  | 1.2 | 1.4 | 1.1 | 1.4 | 1.5 |
| 3- | 0.8 | 0.8 | 0.8 | 0.7 | 0.8 |  | 2.2 | 1.9 | 2.2 | 1.5 | 1.7 |  | 0.8 | 0.8 | 0.5 | 0.4 | 0.4 |  | 1.1 | 1.0 | 1.0 | 1.1 | 1.1 |
| 4- | 0.9 | 0.8 | 0.8 | 0.8 | 0.6 |  | 2.0 | 1.7 | 1.7 | 1.4 | 1.8 |  | 0.3 | 0.2 | 0.4 | 0.5 | 1.0 |  | 1.1 | 1.2 | 1.2 | 1.2 | 1.1 |
| 5- | 0.8 | 0.7 | 1.0 | 0.9 | 1.0 |  | 2.3 | 2.8 | 3.0 | 3.1 | 3.4 |  | 1.0 | 1.2 | 1.0 | 1.2 | 1.6 |  | 0.5 | 0.6 | 0.8 | 0.7 | 0.9 |
| 6- | 0.8 | 0.8 | 0.8 | 0.9 | 1.0 |  | 2.0 | 2.2 | 2.0 | 1.9 | 2.0 |  | 0.5 | 0.6 | 0.6 | 0.3 | 0.4 |  | 0.7 | 0.7 | 0.8 | 1.0 | 1.0 |
| 7- | 0.6 | 0.6 | 0.9 | 1.1 | 1.0 |  | 1.1 | 1.8 | 2.7 | 3.1 | 2.9 |  | 0.2 | 0.9 | 1.0 | 1.5 | 1.9 |  | 0.4 | 0.3 | 0.6 | 0.4 | 0.8 |
| 8- | 1.2 | 1.1 | 1.1 | 1.1 | 1.2 |  | 2.7 | 2.3 | 2.3 | 2.2 | 2.8 |  | 1.1 | 1.1 | 0.6 | 0.7 | 1.0 |  | 0.9 | 1.1 | 1.1 | 1.1 | 1.1 |
| 9 to < 10 | 1.0 | 1.0 | 0.9 | 1.0 | 1.3 |  | 2.4 | 2.3 | 2.2 | 1.8 | 2.3 |  | 1.0 | 0.9 | 0.5 | 0.5 | 0.7 |  | 0.9 | 0.9 | 0.8 | 0.8 | 0.9 |
| 11- | 0.2 | 0.5 | 0.6 | 0.8 | 0.7 |  | 2.0 | 1.5 | 1.5 | 2.0 | 2.5 |  | 0.5 | 0.6 | 0.4 | 0.7 | 1.1 |  | 0.1 | 0.2 | 0.3 | 0.2 | 0.2 |
| 12- | 0.9 | 0.8 | 1.0 | 0.8 | 0.9 |  | 1.8 | 2.1 | 2.0 | 1.7 | 1.8 |  | 0.7 | 0.8 | 0.6 | 0.4 | 0.5 |  | 0.8 | 0.9 | 0.8 | 0.6 | 0.7 |
| 13 to <14 | 1.0 | 1.1 | 0.8 | 0.8 | 1.0 |  | 1.8 | 2.0 | 1.3 | 1.6 | 1.8 |  | 0.7 | 0.6 | 0.8 | 0.4 | 0.4 |  | 1.1 | 1.0 | 0.8 | 0.8 | 1.1 |
| 18- | 1.1 | 1.0 | 1.0 | 0.6 | 0.4 |  | 2.6 | 2.0 | 1.5 | 1.6 | 1.6 |  | 0.9 | 0.9 | 0.8 | 0.2 | 0.2 |  | 0.8 | 0.8 | 0.7 | 0.4 | 0.2 |
| 19 to <20** | 0.9 | 1.1 | 0.9 | 0.6 | 0.7 |  | 1.1 | 2.1 | 1.3 | 1.3 | 0.7 |  | 1.0 | 0.4 | 0.6 | 0.4 | 1.0 |  | 0.8 | 0.9 | 0.6 | 0.6 | 1.1 |
| 23- | 1.0 | 0.6 | 1.1 | 0.7 | 0.4 |  | 3.3 | 2.9 | 1.8 | 2.3 | 1.4 |  | 1.4 | 1.2 | 1.0 | 0.6 | 0.3 |  | 0.7 | 0.4 | 0.6 | 0.3 | -0.2 |
| 24- | 1.03 | 1.1 | 0.9 | 0.5 | 0.6 |  | 2.7 | 2.5 | 1.6 | 1.4 | 1.3 |  | 1.0 | 1.1 | 0.8 | 0.5 | 0.2 |  | 0.6 | 0.6 | 0.5 | 0.3 | 0.5 |
| 25 to <26 | 1.3 | 1.2 | 0.9 | 0.5 | 0.5 |  | 4.1 | 2.8 | 2.4 | 1.4 | 2.5 |  | 1.3 | 0.9 | 0.7 | 0.6 | 0.6 |  | 0.2 | 0.4 | 0.4 | 0.2 | 0.0 |
| * data are presented if sample size > 100 observations. ** the sample size in this age group was 90 or 91. The difference is the percentile values in this study minus the WHO standards | | | | | | | | | | | | | | | | | | | | | | | |

| Table S8. Distribution of weight by length among 2174 infant boys and 2077 infant girls. | | | | | | | | | | | |
| --- | --- | --- | --- | --- | --- | --- | --- | --- | --- | --- | --- |
| Length (cm) | Weight (kg) | | | | | | | | | | |
|  | boys | | | | |  | girls | | | | |
|  | P3 | P10 | P50 | P90 | P97 |  | P3 | P10 | P50 | P90 | P97 |
| 48 | 2.55 | 2.68 | 2.92 | 3.32 | 3.60 |  | 2.53 | 2.69 | 2.97 | 3.44 | 3.65 |
| 49 | 2.60 | 2.78 | 3.15 | 3.60 | 3.84 |  | 2.63 | 2.80 | 3.11 | 3.54 | 3.76 |
| 50 | 2.84 | 3.00 | 3.36 | 3.77 | 4.00 |  | 2.85 | 3.00 | 3.34 | 3.75 | 4.00 |
| 51 | 3.04 | 3.22 | 3.62 | 4.04 | 4.23 |  | 3.06 | 3.23 | 3.63 | 4.15 | 4.49 |
| 52 | 3.20 | 3.39 | 3.85 | 4.45 | 4.80 |  | 3.19 | 3.49 | 3.96 | 4.58 | 4.80 |
| 53 | 3.37 | 3.65 | 4.29 | 5.00 | 5.30 |  | 3.60 | 3.85 | 4.30 | 4.90 | 5.20 |
| 54 | 3.60 | 4.04 | 4.50 | 5.10 | 5.40 |  | 3.80 | 4.00 | 4.50 | 5.00 | 5.40 |
| 55 | 4.00 | 4.20 | 4.80 | 5.40 | 5.60 |  | 4.01 | 4.25 | 4.75 | 5.25 | 5.65 |
| 56 | 4.20 | 4.43 | 5.00 | 5.60 | 6.00 |  | 4.20 | 4.50 | 4.90 | 5.50 | 5.75 |
| 57 | 4.47 | 4.60 | 5.20 | 5.70 | 6.10 |  | 4.50 | 4.65 | 5.15 | 5.80 | 6.40 |
| 58 | 4.60 | 5.00 | 5.50 | 6.10 | 6.75 |  | 4.50 | 4.80 | 5.50 | 6.20 | 6.70 |
| 59 | 4.55 | 5.00 | 5.80 | 6.55 | 7.10 |  | 5.00 | 5.30 | 5.92 | 6.76 | 7.10 |
| 60 | 5.20 | 5.50 | 6.20 | 7.10 | 7.80 |  | 5.30 | 5.60 | 6.30 | 7.05 | 7.75 |
| 61 | 5.70 | 5.95 | 6.50 | 7.50 | 8.00 |  | 5.60 | 5.90 | 6.50 | 7.30 | 7.70 |
| 62 | 5.92 | 6.20 | 6.90 | 7.70 | 8.10 |  | 5.70 | 6.00 | 6.78 | 7.60 | 8.00 |
| 63 | 6.00 | 6.35 | 7.15 | 8.00 | 8.50 |  | 6.00 | 6.13 | 7.00 | 7.90 | 8.40 |
| 64 | 6.20 | 6.54 | 7.45 | 8.20 | 8.60 |  | 6.10 | 6.40 | 7.20 | 8.00 | 8.60 |
| 65 | 6.50 | 6.90 | 7.60 | 8.50 | 9.05 |  | 6.25 | 6.60 | 7.50 | 8.40 | 8.75 |
| 66 | 6.60 | 7.00 | 8.00 | 8.90 | 9.40 |  | 6.50 | 6.85 | 7.80 | 8.70 | 9.15 |
| 67 | 6.85 | 7.35 | 8.10 | 9.05 | 9.50 |  | 6.70 | 7.20 | 8.00 | 9.09 | 9.55 |
| 68 | 7.10 | 7.50 | 8.45 | 9.30 | 9.90 |  | 7.00 | 7.40 | 8.30 | 9.10 | 9.75 |
| 69 | 7.40 | 7.80 | 8.65 | 9.60 | 10.00 |  | 7.20 | 7.55 | 8.50 | 9.50 | 9.90 |
| 70 | 7.50 | 8.00 | 9.00 | 10.00 | 10.50 |  | 7.50 | 7.80 | 8.75 | 9.80 | 10.30 |
| 71 | 7.85 | 8.20 | 9.10 | 10.30 | 11.00 |  | 7.70 | 8.00 | 8.80 | 10.00 | 10.80 |
| 72 | 7.95 | 8.40 | 9.40 | 10.30 | 11.20 |  | 7.80 | 8.10 | 9.00 | 10.20 | 10.75 |
| 73 | 8.10 | 8.50 | 9.55 | 10.60 | 11.35 |  | 8.00 | 8.25 | 9.20 | 10.30 | 10.90 |
| 74 | 8.40 | 8.80 | 9.80 | 11.10 | 12.00 |  | 8.20 | 8.65 | 9.60 | 10.80 | 11.45 |
| 75 | 8.55 | 9.10 | 10.00 | 11.15 | 12.00 |  | 8.10 | 8.75 | 9.80 | 10.80 | 11.60 |
| 76 | 8.70 | 9.30 | 10.20 | 11.35 | 12.00 |  | 8.50 | 9.00 | 10.00 | 11.10 | 11.90 |
| 77 | 9.10 | 9.50 | 10.40 | 11.60 | 12.40 |  | 8.90 | 9.10 | 10.20 | 11.20 | 11.70 |
| 78 | 9.10 | 9.60 | 10.70 | 12.00 | 12.85 |  | 8.90 | 9.45 | 10.40 | 11.70 | 12.50 |
| 79 | 9.30 | 9.80 | 11.00 | 12.00 | 12.60 |  | 9.30 | 9.50 | 10.52 | 11.90 | 12.70 |
| 80 | 9.70 | 10.20 | 11.20 | 12.55 | 13.10 |  | 9.50 | 9.80 | 11.00 | 12.00 | 12.90 |
| 81 | 9.75 | 10.10 | 11.25 | 12.40 | 13.10 |  | 9.31 | 9.90 | 10.95 | 12.20 | 12.95 |
| 82 | 9.70 | 10.30 | 11.50 | 12.60 | 13.10 |  | 9.60 | 10.00 | 11.20 | 12.30 | 13.00 |
| 83 | 10.00 | 10.45 | 11.60 | 13.00 | 13.60 |  | 9.80 | 10.20 | 11.30 | 12.40 | 13.10 |
| 84 | 10.20 | 10.50 | 11.80 | 12.90 | 13.30 |  | 9.90 | 10.30 | 11.40 | 12.60 | 13.10 |
| 85 | 10.50 | 10.90 | 12.00 | 13.40 | 14.20 |  | 10.15 | 10.60 | 11.80 | 13.00 | 13.80 |
| 86 | 10.50 | 11.20 | 12.20 | 13.50 | 14.20 |  | 10.20 | 10.70 | 12.00 | 13.00 | 14.20 |
| 87 | 10.70 | 11.30 | 12.40 | 13.90 | 14.60 |  | 10.65 | 11.13 | 12.20 | 13.20 | 14.10 |
| 88 | 11.00 | 11.40 | 12.70 | 13.80 | 14.40 |  | 10.75 | 11.20 | 12.40 | 13.70 | 14.50 |
| 89 | 10.80 | 11.60 | 12.95 | 14.10 | 14.60 |  | 10.80 | 11.50 | 12.65 | 13.90 | 14.80 |
| 90 | 11.70 | 12.00 | 13.10 | 14.40 | 15.20 |  | 11.10 | 11.85 | 13.20 | 14.50 | 15.00 |
| 91 | 11.80 | 12.20 | 13.50 | 15.05 | 15.40 |  | 11.50 | 11.80 | 13.10 | 14.65 | 15.50 |
| 92 to <93** | 12.20 | 12.60 | 13.65 | 15.10 | 16.00 |  | 12.00 | 12.50 | 13.45 | 15.20 | 16.40 |
| * data are presented if sample size > 100 observations. ** the sample size in this group was 66 in girls. | | | | | | | | | | | |

| Table S9. The difference between the percentile values of the raw data of among 2174 infant boys and 2077 infant girls and the corresponding WHO standards. | | | | | | | | | | | |
| --- | --- | --- | --- | --- | --- | --- | --- | --- | --- | --- | --- |
|  | Weight difference (The present study – WHO), kg | | | | | | | | | | |
|  | boys | | | | |  | girls | | | | |
| Length (cm) | P3 | P10 | P50 | P90 | P97 |  | P3 | P10 | P50 | P90 | P97 |
| 48 | 0.05 | 0.08 | 0.02 | 0.02 | 0.1 |  | 0.03 | -0.01 | -0.03 | 0.14 | 0.15 |
| 49 | -0.1 | -0.02 | 0.05 | 0.1 | 0.14 |  | -0.07 | 0 | -0.09 | -0.06 | -0.04 |
| 50 | 0.04 | 0 | 0.06 | 0.07 | 0 |  | 0.05 | 0 | -0.06 | -0.05 | 0 |
| 51 | 0.04 | 0.02 | 0.12 | 0.04 | 0.03 |  | 0.06 | 0.03 | 0.03 | 0.15 | 0.19 |
| 52 | 0 | -0.01 | 0.05 | 0.25 | 0.3 |  | -0.01 | 0.09 | 0.16 | 0.28 | 0.3 |
| 53 | -0.03 | 0.05 | 0.29 | 0.5 | 0.6 |  | 0.2 | 0.25 | 0.3 | 0.4 | 0.4 |
| 54 | 0 | 0.24 | 0.2 | 0.3 | 0.4 |  | 0.2 | 0.2 | 0.2 | 0.2 | 0.3 |
| 55 | 0.1 | 0.1 | 0.3 | 0.3 | 0.2 |  | 0.11 | 0.15 | 0.25 | 0.15 | 0.25 |
| 56 | 0.1 | 0.13 | 0.2 | 0.2 | 0.3 |  | 0.1 | 0.2 | 0.1 | 0.1 | -0.05 |
| 57 | 0.07 | 0 | 0.1 | 0 | 0.1 |  | 0.2 | 0.15 | 0.05 | 0.1 | 0.3 |
| 58 | 0 | 0.1 | 0.1 | 0.1 | 0.35 |  | 0 | 0 | 0.1 | 0.2 | 0.3 |
| 59 | -0.35 | -0.1 | 0.1 | 0.15 | 0.4 |  | 0.2 | 0.3 | 0.32 | 0.46 | 0.4 |
| 60 | 0.1 | 0.1 | 0.2 | 0.4 | 0.8 |  | 0.3 | 0.4 | 0.4 | 0.45 | 0.75 |
| 61 | 0.3 | 0.35 | 0.2 | 0.5 | 0.6 |  | 0.4 | 0.4 | 0.4 | 0.4 | 0.4 |
| 62 | 0.32 | 0.3 | 0.4 | 0.4 | 0.4 |  | 0.3 | 0.3 | 0.38 | 0.4 | 0.4 |
| 63 | 0.2 | 0.25 | 0.35 | 0.4 | 0.5 |  | 0.4 | 0.23 | 0.4 | 0.4 | 0.5 |
| 64 | 0.2 | 0.24 | 0.45 | 0.4 | 0.4 |  | 0.3 | 0.3 | 0.3 | 0.3 | 0.4 |
| 65 | 0.2 | 0.3 | 0.3 | 0.4 | 0.55 |  | 0.25 | 0.3 | 0.4 | 0.4 | 0.25 |
| 66 | 0.1 | 0.2 | 0.5 | 0.5 | 0.6 |  | 0.3 | 0.35 | 0.5 | 0.5 | 0.45 |
| 67 | 0.15 | 0.35 | 0.4 | 0.45 | 0.4 |  | 0.3 | 0.5 | 0.5 | 0.59 | 0.55 |
| 68 | 0.2 | 0.3 | 0.45 | 0.4 | 0.6 |  | 0.4 | 0.5 | 0.6 | 0.4 | 0.55 |
| 69 | 0.3 | 0.4 | 0.45 | 0.5 | 0.4 |  | 0.5 | 0.45 | 0.5 | 0.5 | 0.4 |
| 70 | 0.3 | 0.4 | 0.6 | 0.6 | 0.6 |  | 0.6 | 0.5 | 0.55 | 0.6 | 0.6 |
| 71 | 0.45 | 0.4 | 0.5 | 0.7 | 0.9 |  | 0.6 | 0.5 | 0.4 | 0.6 | 0.8 |
| 72 | 0.35 | 0.4 | 0.5 | 0.4 | 0.8 |  | 0.5 | 0.5 | 0.4 | 0.6 | 0.55 |
| 73 | 0.3 | 0.3 | 0.45 | 0.5 | 0.65 |  | 0.6 | 0.45 | 0.4 | 0.4 | 0.5 |
| 74 | 0.4 | 0.4 | 0.5 | 0.7 | 1.1 |  | 0.6 | 0.65 | 0.6 | 0.7 | 0.75 |
| 75 | 0.35 | 0.5 | 0.5 | 0.55 | 0.8 |  | 0.3 | 0.55 | 0.7 | 0.5 | 0.7 |
| 76 | 0.4 | 0.6 | 0.5 | 0.55 | 0.6 |  | 0.6 | 0.7 | 0.7 | 0.6 | 0.8 |
| 77 | 0.6 | 0.6 | 0.5 | 0.6 | 0.8 |  | 0.8 | 0.6 | 0.7 | 0.5 | 0.4 |
| 78 | 0.4 | 0.5 | 0.6 | 0.8 | 1.05 |  | 0.7 | 0.75 | 0.7 | 0.8 | 1 |
| 79 | 0.5 | 0.6 | 0.7 | 0.6 | 0.5 |  | 0.9 | 0.7 | 0.62 | 0.8 | 0.9 |
| 80 | 0.7 | 0.8 | 0.8 | 0.95 | 0.8 |  | 0.9 | 0.8 | 0.9 | 0.7 | 0.9 |
| 81 | 0.65 | 0.5 | 0.65 | 0.5 | 0.6 |  | 0.51 | 0.7 | 0.65 | 0.6 | 0.75 |
| 82 | 0.4 | 0.5 | 0.7 | 0.5 | 0.4 |  | 0.7 | 0.6 | 0.7 | 0.5 | 0.5 |
| 83 | 0.5 | 0.45 | 0.6 | 0.7 | 0.6 |  | 0.7 | 0.6 | 0.6 | 0.3 | 0.3 |
| 84 | 0.5 | 0.3 | 0.5 | 0.4 | 0.1 |  | 0.6 | 0.5 | 0.4 | 0.3 | 0 |
| 85 | 0.6 | 0.5 | 0.5 | 0.6 | 0.7 |  | 0.65 | 0.6 | 0.6 | 0.4 | 0.5 |
| 86 | 0.4 | 0.6 | 0.5 | 0.4 | 0.5 |  | 0.4 | 0.4 | 0.5 | 0.1 | 0.6 |
| 87 | 0.4 | 0.5 | 0.4 | 0.6 | 0.6 |  | 0.65 | 0.63 | 0.5 | 0 | 0.2 |
| 88 | 0.4 | 0.3 | 0.5 | 0.2 | 0.1 |  | 0.55 | 0.5 | 0.4 | 0.2 | 0.3 |
| 89 | 0 | 0.3 | 0.45 | 0.2 | 0 |  | 0.4 | 0.6 | 0.45 | 0.2 | 0.3 |
| 90 | 0.7 | 0.5 | 0.4 | 0.3 | 0.3 |  | 0.5 | 0.65 | 0.7 | 0.5 | 0.2 |
| 91 | 0.6 | 0.5 | 0.5 | 0.65 | 0.3 |  | 0.7 | 0.4 | 0.4 | 0.35 | 0.4 |
| 92 to <93** | 0.8 | 0.7 | 0.45 | 0.5 | 0.6 |  | 1 | 0.9 | 0.45 | 0.6 | 1 |
| * data are presented if sample size > 100 observations. ** the sample size in this group was 66 in girls.  The difference is the raw percentile values in this study minus the WHO standards. | | | | | | | | | | | |
